# Whole-genome sequencing reveals the evolutionary trajectory of HBV-related hepatocellular carcinoma early recurrence

**DOI:** 10.1038/s41392-021-00838-3

**Published:** 2022-01-26

**Authors:** Shao-Lai Zhou, Zheng-Jun Zhou, Cheng-Li Song, Hao-Yang Xin, Zhi-Qiang Hu, Chu-Bin Luo, Yi-Jie Luo, Jia Li, Zhi Dai, Xin-Rong Yang, Ying-Hong Shi, Zheng Wang, Xiao-Wu Huang, Jia Fan, Jian Zhou

**Affiliations:** 1 Department of Liver Surgery and Transplantation, Zhongshan Hospital, Fudan University, Shanghai, 200032 China; 2 Liver Cancer Institute, Zhongshan Hospital, Fudan University, Shanghai, 200032 China; 3 Novogene Bioinformatics Institute, Beijing, 100083 China; 4 Institute of Cancer Stem Cell, Dalian Medical University, Dalian, Liaoning 116044 China; 5 State Key Laboratory of Genetic Engineering, Fudan University, Shanghai, 200032 China

**Keywords:** Cancer genomics, Gastrointestinal cancer

## Abstract

Patients with hepatocellular carcinoma (HCC) have poor long-term survival following curative resection because of the high rate of tumor early recurrence. Little is known about the trajectory of genomic evolution from primary to early-recurrent HCC. In this study, we performed whole-genome sequencing (WGS) on 40 pairs of primary and early-recurrent hepatitis B virus (HBV)-related HCC tumors from patients who received curative resection, and from four patients whose primary and recurrent tumor were extensively sampled. We identified two recurrence patterns: de novo recurrence (18/40), which developed genetically independently of the primary tumor and carried different HCC drivers, and ancestral recurrence (22/40), which was clonally related to the primary tumor and progressed more rapidly than de novo recurrence. We found that the recurrence location was predictive of the recurrence pattern: distant recurrence tended to display the de novo pattern, whereas local recurrence tended to display the ancestral pattern. We then uncovered the evolutionary trajectories based on the subclonal architecture, driver-gene mutations, and mutational processes observed in the primary and recurrent tumors. Multi-region WGS demonstrated spatiotemporal heterogeneity and polyclonal, monophyletic dissemination in HCC ancestral recurrence. In addition, we identified recurrence-specific mutations and copy-number gains in *BCL9*, leading to WNT/β-catenin signaling activation and an immune-excluded tumor microenvironment, which suggests that BCL9 might serve as a new therapeutic target for recurrent HCC. Collectively, our results allow us to view with unprecedented clarity the genomic evolution during HBV-related HCC early recurrence, providing an important molecular foundation for enhanced understanding of HCC with implications for personalized therapy to improve patient survival.

## Introduction

The incidence and mortality rates of hepatocellular carcinoma (HCC), one of the most prevalent types of cancer, have increased in recent years.^1^ Over the past decade, we have expanded our understanding of HCC pathogenesis at the molecular level. Following technological advances, several studies revealed the genetic landscape of alterations that underlie liver carcinogenesis.^2–7^ Previously, we and others delineated the genomic events that characterize Chinese HCCs.^8,9^

Only a minority of HCCs are diagnosed at early stages, when curative treatments are feasible.^10,11^ Advances in surgical techniques and perioperative management have improved the survival of patients with HCC; however, the high rate of tumor recurrence limits long-term survival even after surgical resection.^12–15^ HCC recurrence after resection can be classified as early, occurring within weeks or months, or late, occurring >2 years after resection.^16^ Early recurrence accounts for nearly 70% of all HCC recurrence and is attributable either to micrometastases that occur in the liver outside of the treated area or to incomplete treatment of the primary tumor. Late recurrence, on the other hand, is attributable to new cancers, or primary lesions that develop independently of the previously treated lesion.^16^ To date, no direct evidence is available to discriminate between those two recurrence mechanisms. Molecular profiling of HCC has typically focused on primary tumors and therefore has not identified any general patterns of evolution between primary and recurrence, leaving a number of unanswered questions with important biological and clinical implications.

We performed whole-genome sequencing (WGS) on 40 pairs of primary and recurrent hepatitis B virus (HBV)-related HCC tumors obtained at primary diagnosis and at the time of early recurrence after curative resection. We compared the genetic profiles of the primary and early-recurrent tumors to determine: (1) whether the recurrent tumors are derived from micrometastases of the primary tumors, (2) how close the genetic relation is between the primary tumors and the recurrent tumors, (3) whether there are differences in mutational processes between the primary and recurrent tumors, (4) if recurrent tumors are clonally derived from primary tumors, how the recurrent tumors evolve from the primary tumors. Because the survival of patients with recurrent HCC is poor, it is particularly important to establish whether HCC recurrence is driven by newly emerging driver mutations, which might offer opportunities for personalized therapy.

## Results

### The genomic landscape of 40 matched pairs of primary and early-recurrent HCCs

**Fig. 1:**
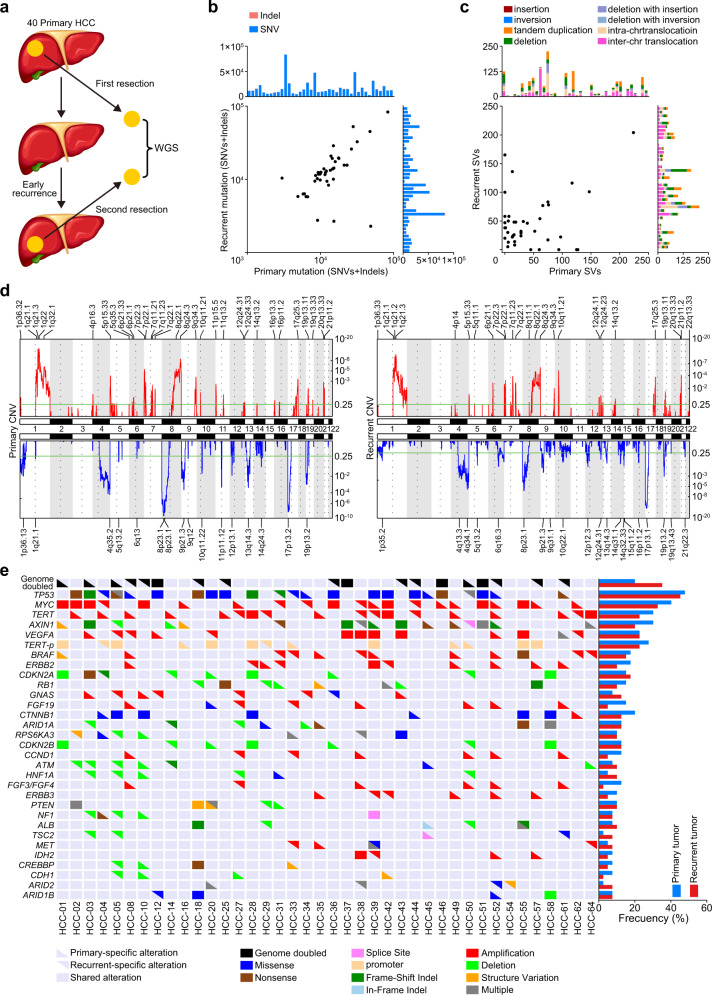
Genomic landscape of 40 pairs of primary and early-recurrent HCCs. **a** A concise diagram of the sequencing research in the present HCC genomic study. **b** The number of somatic mutations (SNVs + indels) in the whole genome across 40 pairs of primary and early-recurrent HCCs. **c** The number of structural variations (SVs) in 40 pairs of primary and early-recurrent HCCs, including insertions, inversions, tandem duplications, deletions, and inter- and intra-chromosomal translocations. **d** GISTIC analysis revealed the whole-genome distribution of copy-number alterations in paired primary (left panel) and early-recurrent (right panel) HCCs. GISTIC *q*-values (*y*-axis) for amplifications (upper, red) and deletions (lower, blue) are plotted across the genome (*x*-axis). **e** The genomic alteration of driver genes in 40 pairs of primary and early-recurrent HCCs, identified through whole-genome sequencing. Drivers present in ≥4 patients are shown. All mutations (SNVs + indels) were validated by Sanger sequencing

We performed WGS of 40 matched pairs of primary HCC and early-recurrent HCC and matched non-cancerous liver samples from the same patients (Fig. 1a). The average sequencing depth was 54.3-fold for the primary tumors, 54.1-fold for the recurrent tumors, and 36.4-fold for the normal tissues (Supplementary Table 2). We identified a total of 667,790 somatic single-nucleotide variations (SNVs) and indels in the primary tumors (2,990–83,437 per tumor, mean: 16695). The numbers were comparable in the recurrent tumors, which had 653,974 somatic SNVs and indels (2,285–83,312 per tumor, mean: 16349; Fig. 1b; Supplementary Table 3). Sanger sequencing of 1560 randomly selected somatic coding mutations showed that the true-discovery rate was high (95.3%).

We identified 31 amplified segments in the primary tumors, which harbored several known oncogenes including *CCND1*, *TERT*, and *MYC*. We also identified 16 lost segments in the primary tumors, which harbored tumor suppressors including *TP53* (17p13), *RB1* (13q14), and *CDKN2A* (9q21). In the recurrent tumors, we identified 25 amplified segments and 20 lost segments (Fig. 1d; Supplementary Table 4). Additionally, we detected abundant genomic structural variations (SVs), with averages of 43.8 SVs (range: 0–224) per primary tumor and 47.8 SVs (range: 0–204) per recurrent tumor. Those SVs comprised 839 deletions, 37 deletions with inversion, 335 deletions with insertion, 692 tandem duplications, 8 insertions, 5 inversions, and 1747 intra-chromosomal or inter-chromosomal translocations (Fig. 1c; Supplementary Table 5). We detected 44 HBV integration breakpoints in 23 (57.5%) of the primary tumor samples and 45 HBV integration breakpoints in 24 (60%) of the recurrent tumor samples, including recurrent HBV integration events in *TERT*, a well-known HCC driver gene (Supplementary Table 6).

Integrative analysis of the somatic mutations (nonsynonymous SNVs and indels), CNVs, and SVs revealed several common types of HCC driver-gene alterations, which had comparable numbers in the primary and recurrent tumors, including *TP53* mutation, *MYC* amplification, and *TERT* amplification or promoter mutation (Fig. 1e).

### Recurrence patterns in HCC early recurrence after curative resection

After original mutation calling, we found that the patients could be clearly separated into two groups based on the proportions of shared somatic mutations between the primary and recurrent tumors: patients with a low rate of shared mutations between primary and recurrent tumors (mean = 0.8%, range: 0.2–1.5%) and patients with a high rate of shared mutations (mean = 51.9%, range: 22.1–75.2%; *P* = 7.18 × 10^−8^, Mann−Whitney U test; Supplementary Fig. 2a, b; Supplementary Table 7). We also analyzed shared mutation rates between randomly selected tumor pairs (780 simulated pairs) from WGS data of 40 independent primary HCC samples to determine the occurrence of the same alteration in independent samples detected by the same pipeline. The shared mutation rates among independent tumor pairs ranged from 0.005–0.20%. Considering that somatic mutations rarely occur at the same position in the human genome multiple times as independent events,^17^ our findings suggest that the recurrent tumors that shared a high rate of mutations with their respective primary tumors were probably derived from the original clones. We considered those tumors to be instances of ancestral recurrence. Conversely, we considered the recurrent tumors that shared low rates of mutations with their respective primary tumors to be genetically independent of the primary tumors and hence to be instances of de novo recurrence.

**Fig. 2:**
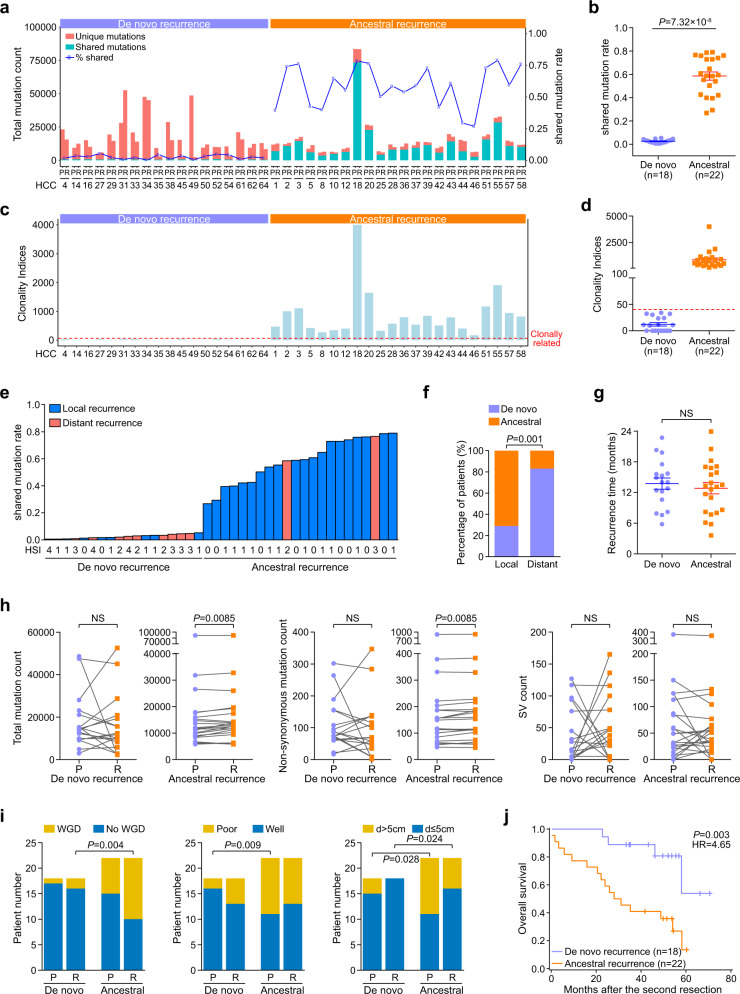
Recurrence patterns during HCC early recurrence after curative resection. **a** Numbers of somatic mutations (SNVs + Indels) and frequency of shared mutations at the whole-genome level between primary tumors and recurrent tumors across 40 patients with HCC after force calling. **b** The proportion of shared mutations in patients with de novo recurrence and ancestral recurrence. **c**, **d** Clonality indices for the 40 cases of HCC analyzed in our study. Red dotted lines indicate the cut-off value to define clonal relatedness. **e**, **f** The proportion of shared mutations and its relationship to the recurrence location. **g** Comparison of recurrence time between patients with de novo recurrence and patients with ancestral recurrence. **h** Comparison of total somatic mutations (left panel), non-synonymous mutations (middle panel), and SVs (right panel) between primary tumors and recurrent tumors in patients with de novo recurrence or ancestral recurrence. **i** Comparison of whole-genome doubling (WGD), tumor cell differentiation, and tumor size between patients with de novo recurrence and patients with ancestral recurrence in primary or recurrent tumors. **j** Kaplan−Meier analysis revealed different overall survival rates between the two recurrence patterns after the second resection

During cancer evolution, copy number losses may result in the loss of mutations in the affected regions, resulting in the appearance of clusters of mutations uniquely in samples that are unaffected by the copy-number loss. In addition, some mutations that are present in multiple samples might only be called in a subset of those samples because of low mutant-allele frequencies.^18^ Those factors might affect the accuracy of our classification of recurrence patterns. Therefore, in order to make sure that our classification was valid, we excluded mutations that were identified in one sample but were located in a region affected by CNV loss in other samples in which they did not appear (CNV drop) and then re-called the allele frequencies of all the mutations that were found in any of the samples from each patient (force calling). After the CNV drop and force calling, we observed that the proportions of mutations that were shared by the primary tumors and the recurrent tumors increased in all of the patients, as expected. There was also, however, clear separation between two groups of patients in terms of the proportion of mutations that were shared between the primary and recurrent tumors, with one group having a very low proportion of shared mutations (mean = 2.6%, range: 0.6–5.3%) and the other group having a much higher proportion (mean = 58.6%, range: 26.8–79%; *P* = 7.32 × 10^−8^, Mann−Whitney U test; Fig. 2a, b; Supplementary Table 7).

We also used a conservative analytic approach^19^ to estimate the clonality indices of the tumors on the basis of nonsynonymous SNVs and indels, which showed the likelihood of two tumors sharing mutations that were not expected to have co-occurred by chance. The 40 patients were clearly separated into two groups based on a calculated threshold (36.59): those with clonality indices below the threshold (mean = 12.24, range: 0–34.11) and those with clonality indices above the threshold (mean = 883.2, range: 167.2–4007; Fig. 2c, d), which was consistent with our aforementioned grouping results. Thus, we identified two patterns of HCC early recurrence: 45% (18 of 40) of the patients experienced de novo recurrence, in which the recurrent tumors were genetically independent of the primary tumors, whereas 55% (22 of 40) of the patients experienced ancestral recurrence, in which the recurrent tumors were clonally related to the primary tumors. In the second pattern, ancestral recurrence, the recurrent tumors were most likely derived from intra-hepatic dissemination of the primary tumors (Supplementary Table 7; Supplementary Fig. 3).

### Recurrence location, but not recurrence time, was predictive of the type of recurrence

Next, we explored the association between the recurrence location and the recurrence patterns. We classified the 40 pairs of primary and recurrent tumors as local recurrence (28 of 40), with tumor hepatic segment interval (HSI) 0 or 1, or distant recurrence (12 of 40), with tumor HSI from 2 to 4. In the 28 patients with local recurrence, 20 of the recurrent tumors were ancestral recurrences, whereas in the 12 patients with distant recurrence, only 2 of the recurrent tumors were ancestral recurrences (*P* = 0.001, chi-square test; Fig. 2e, f; Supplementary Fig. 3; Supplementary Table 8). Those results suggest that local recurrence tends to be ancestral, whereas distant recurrence is prone to be de novo. The association between the recurrence location and the recurrence patterns was not absolute in our sample, however. Among the 11 patients with HSI 0, two patients experienced de novo recurrence, suggesting that although the recurrent tumors were located in the same hepatic segment as the primary tumors, they were still new primary tumors. On the other hand, among the eight patients with HSI > 2, one patient experienced ancestral recurrence, with the primary tumor in hepatic segment VI and the recurrent tumor in hepatic segment III, suggesting that intra-hepatic metastases of primary tumors can disseminate across nearly the whole liver (Supplementary Table 8). We also tested the association between recurrence patterns and recurrence time. The results showed no association between them, which suggested that recurrence time is not predictive of early recurrence patterns in HCC (Fig. 2g).

### Comparison of genomic and clinical characteristics between de novo and ancestral recurrence

To characterize features of genomic alterations in HCC patients with de novo or ancestral recurrence, we analyzed nonsynonymous SNVs and indels and SVs. We found that the proportions of nonsynonymous mutations that were shared between the primary and recurrent tumors were clearly different between the patients with de novo recurrence and those with ancestral recurrence (mean: 0.8% vs. 66.4%, *P* = 6.51 × 10^−8^, Mann−Whitney U test; Supplementary Fig. 2c, d). In the SV analysis, there were no shared SVs between the primary and recurrent tumors in the patients with de novo recurrence. By contrast, all except four of the patients with ancestral recurrence had SVs shared between the primary and recurrent tumors, and those four patients had no SVs detected in one of their tumors (Supplementary Fig. 2e, f). Those results suggest that nonsynonymous mutations and SVs that are shared between primary and recurrent tumors can serve as an alternative marker for the classification of HCC early recurrence patterns. We also found no shared HBV integration breakpoints in the 7 of 18 patients with de novo recurrence who had HBV integration events in both primary and recurrent tumors. In the 11 of 22 patients with ancestral recurrence who had HBV integration events in both primary and recurrent tumors, we identified at least one shared HBV integration breakpoint in each of the 11 patients (Supplementary Table 6).

In the patients with de novo recurrence, we observed considerable variability in the numbers of mutations and SVs between the primary and recurrent tumors. In the patients with ancestral recurrence, that variability was smaller, and the recurrent tumors always exhibited a higher number of mutations (total mutations and nonsynonymous mutations) than the primary tumors (paired *t* test, Fig. 2h, Supplementary Figs. 4, 5). Those results suggest that although the primary and recurrent tumors had comparable numbers of mutations across all 40 patients, ancestrally recurrent tumors usually obtained extra mutations during their evolution.

Tumor ploidy abnormalities are a hallmark of cancer and have an important impact on the evolution and outcomes of different cancers.^20^ A pan cancer study revealed that the median ploidy of tumors that underwent whole-genome doubling (WGD) was 3.3 [interquartile range (IQR): 2.9–3.8].^20^ In our study, tumors were considered to have undergone WGD if their ploidy was greater than 2.9. At this threshold, we identified WGD in 8 of the 40 primary tumors and in 14 of the 40 recurrent tumors (Supplementary Table 9). Specifically, WGD was enriched in the patients with ancestral recurrence, especially in the recurrent tumors (12/22 vs. 2/18, *P* = 0.004, chi-square test). Those results suggest that WGD in HCC might be a driver of ancestral recurrence (Fig. 2i).

Next, we correlated clinical and pathologic characteristics with recurrence patterns in all 40 patients. The ancestral recurrence patients tended to have poor cell differentiation and larger tumors in primary HCC, and also developed larger tumors in recurrent HCC than those in the de novo recurrence patients (Fig. 2i). Kaplan−Meier survival analysis showed that the survival rate after the second curative resection was lower among the patients with ancestral recurrence than among those with de novo recurrence (Fig. 2j). Univariate and multivariate analyses revealed that the recurrence pattern was an independent prognostic indicator for patient survival after the second resection (Supplementary Table 10). Those results suggest that ancestral recurrence usually progresses rapidly, probably because of micrometastases that occur before the first resection.

### The evolution of HCC early recurrence at the whole-genome level

**Fig. 3:**
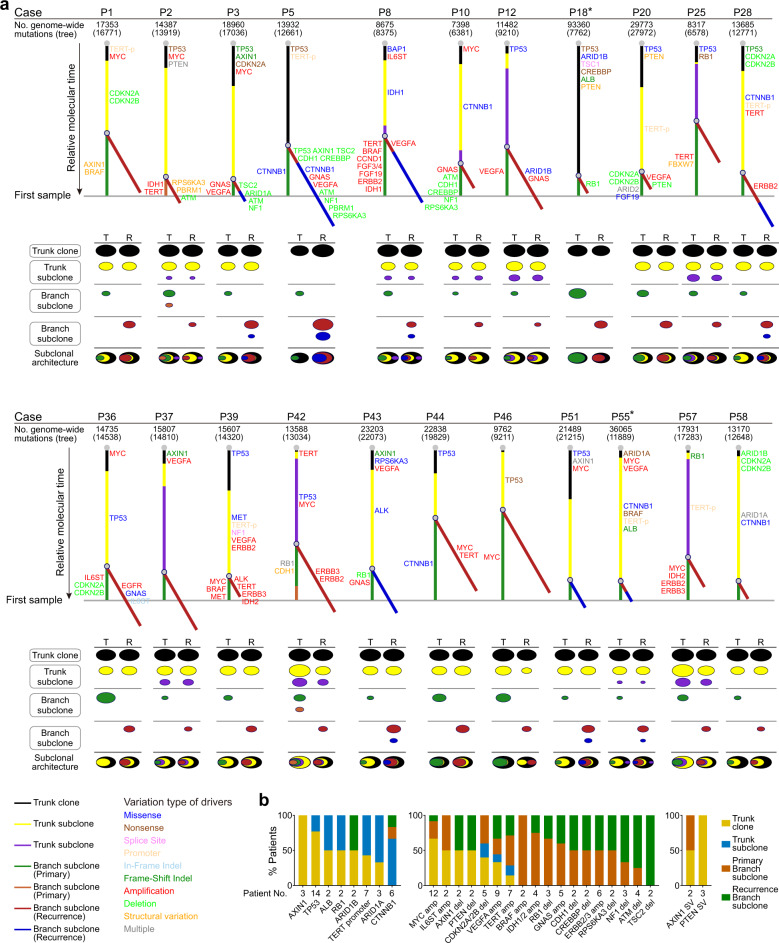
Phylogenetic trees and subclonal architectures underlying the evolutionary trajectory of tumor early recurrence in 22 HCCs with ancestral recurrence. **a** Each tree and corresponding subclonal architecture represents an individual patient. Trees were derived from genome-wide somatic mutations (SNVs + indels) based on subclonal architectures. The numbers of all somatic mutations per patient are labeled above the tree (the numbers of somatic mutations involved in constructing the phylogenetic trees are labeled in brackets). The asterisk indicates that trees and subclonal architectures were derived from somatic mutations in genic region. Line lengths reflect the proportion of clustered somatic mutations attributed to that clone or subclone. The whole tree is scaled to the maximum length of a tree that would be inferred from mutations identified in the primary tumor. In the subclonal architecture panel, the diameter of each oval with color is proportional to the estimated CCF, which reflects the proportion of cells in that sample that contain the mutations that constitute the same color of the relevant phylogenetic tree. **b** The relative fraction of patients in whom the indicated somatic alterations of drivers occupied the trunk clone, trunk subclone, or branch subclone of their respective phylogenetic trees. The numbers of patients with each of the indicated somatic alterations are labeled

**Fig. 4:**
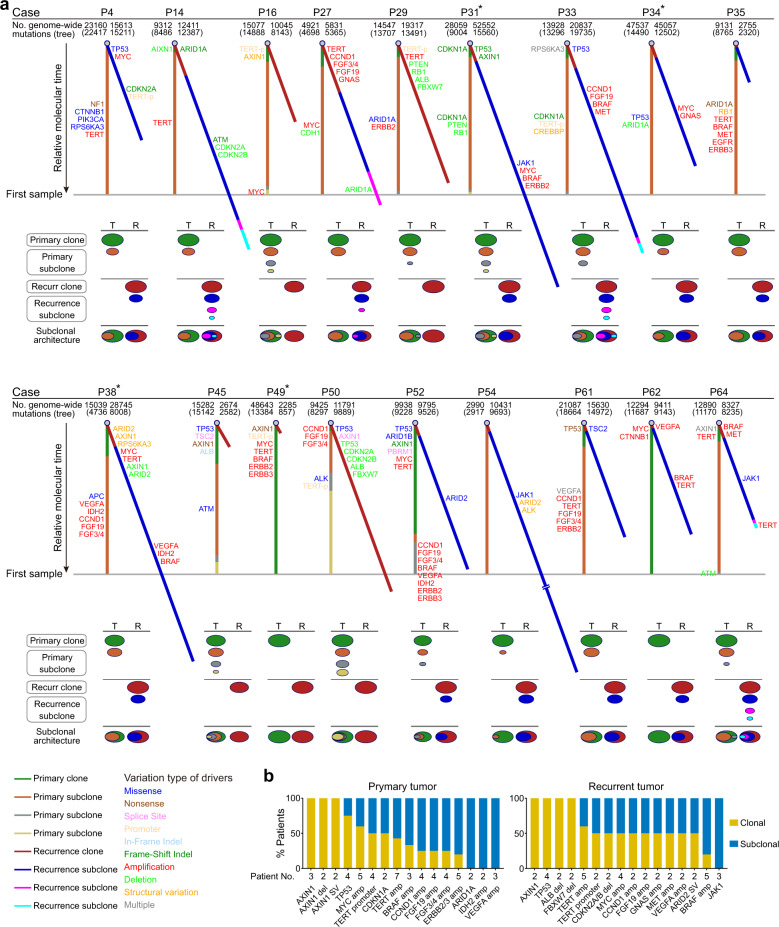
Phylogenetic trees and subclonal architectures underlying the evolutionary trajectory of tumor early recurrence in 18 HCCs with de novo recurrence. **a** Each tree and corresponding subclonal architecture represents an individual patient. Trees were derived from genome-wide somatic mutations (SNVs + indels) based on subclonal architectures. The numbers of all somatic mutations per patient are labeled above the tree (the numbers of somatic mutations involved in constructing the phylogenetic trees are labeled in brackets). The asterisk indicates that trees and subclonal architectures were derived from somatic mutations in genic region. Line lengths reflect the proportion of clustered somatic mutations attributed to that clone or subclone. The whole tree is scaled to the maximum length of a tree that would be inferred from mutations identified in the primary tumor. In the subclonal architecture panel, the diameter of each oval with color is proportional to the estimated CCF, which reflects the proportion of cells in that sample that contain the mutations that constitute the same color of the relevant phylogenetic tree. **b** The relative fraction of patients in whom the indicated somatic alterations of drivers occupied the clone or subclone of their respective phylogenetic trees. The numbers of patients with each of the indicated somatic alterations are labeled

To infer the evolutionary trajectory of HCC early recurrence after curative resection, we constructed subclonal architectures and phylogenetic trees to represent the disease progression of the primary and recurrent tumors in each patient based on mutations (SNVs and indels) identified at the whole-genome level. We grouped the trees according to scenario: ancestral recurrence or de novo recurrence. Although there was a very low proportion of shared mutations between primary tumors and de novo recurrent tumors, nearly all of the shared mutations were subclones (date not shown) according to the cancer cell fraction (CCF) results calculated by pyclone,^21^ and none were in driver genes (Fig. 4). Those results further support that the recurrent tumors developed independently from the primary tumors in the patients with de novo recurrence. Therefore, we constructed phylogenetic trees separately for the primary and recurrent tumors in the patients with de novo recurrence (Figs. 3, 4; Supplementary Figs. 6, 7; Supplementary Tables 11 and 12).

In the patients with ancestral recurrence, we identified an average of 3.4 distinct mutation clusters (group sets of somatic mutations with shared CCFs) per primary tumor and 3.6 distinct clusters per recurrent tumor (Fig. 3a). We divided the trunk mutations into different clones or subclones according to their estimated CCFs. The mutations in the top of the trunk were clonal mutations, and the others were subclonal mutations with decreasing CCFs downwards along the trunk of the tree. A distinct feature was that nearly all of the patients had one or more trunk mutation clusters presented subclonally in both their primary tumor and their recurrent tumor, with the recurrence-branch subclones always descended from the trunk subclonal mutations. That indicated a possibility of polyclonal metastatic seeding in HCC ancestral recurrence, which was further demonstrated by our multi-region WGS (see the results below). Furthermore, in all 20 patients who carried mutations presented trunk subclone, we observed significantly higher mutation numbers in the trunk subclone than in the trunk clone (6886 ± 3241 vs. 1779 ± 1933, 2.05 × 10^−8^, paired *t* test), which might suggest that tumors needed more time to acquire invasive ability to disseminate intra-hepatically than to progress locally. In addition, 7 of 22 (31.8%) recurrent tumors, but only 2 of 22 (9%) primary tumors, had two branch subclones, which suggested enrichment of ITH in the recurrent tumors.

We then mapped the drivers to the phylogenetic trees (Fig. 3a). We observed that most mutation drivers (SNV or indel) presented trunk clones or subclones and had comparable CCFs between primary and recurrent tumors (Supplementary Fig. 8), whereas over half of the CNV drivers were mapped to branch subclones. That suggests that SNVs/indels precede CNVs as oncogenic drivers of intra-hepatic dissemination during HCC early recurrence. This may be due to the progressive accumulation of somatic mutations, often tumor suppressor gene (TSG) mutations, that lead to genomic instability and, subsequently, oncogenic amplifications or TSG deletions.

We then deduced the approximate sequential order in which somatic alterations evolve during HCC ancestral recurrence by calculating how often a given alteration resided on the trunk (clonal or subclonal) versus a branch of each evolutionary tree (Fig. 3b). That analysis confirmed that *AXIN1* and *TP53* mutations were the earliest mutations to evolve, followed by *TERT* promoter, *CTNNB1*, or *ARID1A* mutations, as well as amplifications of *MYC* or *VEGFA* or deletions of *CDKN2A/2B* and *PTEN*. Amplifications affecting *BFAF* and *IDH1/2* were mostly restricted to the primary tumors at later time points, whereas *ATM* or *TSC2* deletions were acquired by recurrence subclones, suggesting that those further events of genomic evolution might abet the growth of the metastatic clone in its new niche. Takeda et al found that *TERT* promoter mutations are required for the early stages of hepatocarcinogenesis.^22^ Our results suggest that *TERT* promoter mutations follow *AXIN1* and *TP53* mutations, although they are also identified as trunk events.

We identified an average of 2.6 distinct mutation clusters per primary tumor and 2.1 distinct clusters per recurrent tumor in the patients with de novo recurrence (Fig. 4a). Compared with ancestral recurrence, de novo recurrence was associated with a lack of *CTNNB1* mutation and enrichment of *CCND1-FGF19* amplification, especially in the primary tumors (Figs. 3b, 4b). Furthermore, we observed that the alterations involving HCC driver genes were entirely different between the primary and recurrent tumors within each patient with de novo recurrence. Nevertheless, most driver alterations had similar frequencies and clonal distributions between the primary and recurrent tumors across all of the patients with de novo recurrence: alterations in *AXIN1* and *TP53* always took place early in the evolution of the tumor, whereas *TERT* promoter mutations and amplification were often acquired subsequently, and CNVs affecting *CCND1*-*FGF19* and *VEGFA* occurred at later points in the evolutionary cascade.

### The mutational spectrum and signatures in primary and early-recurrent HCC

**Fig. 5:**
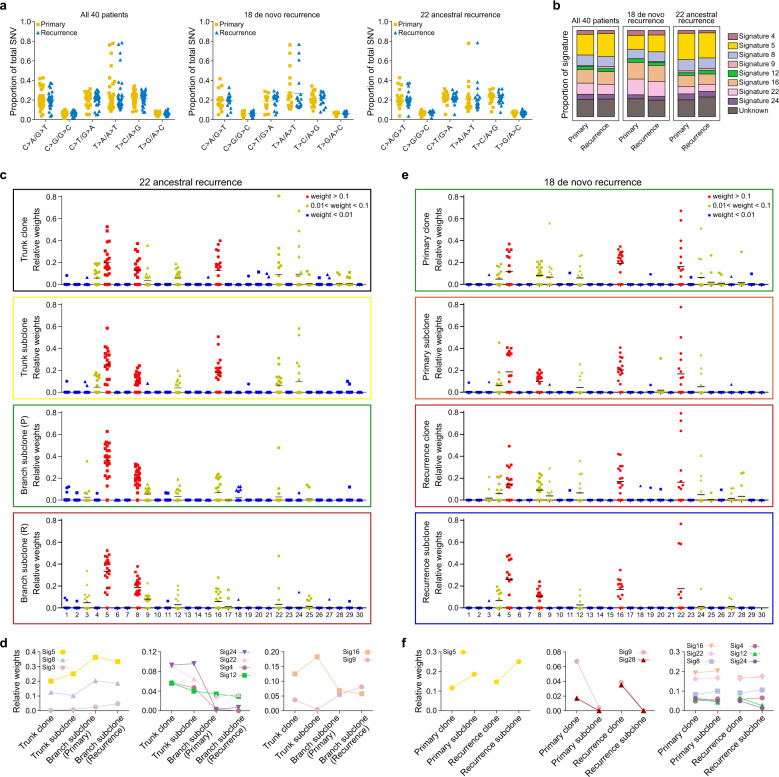
Mutational spectrum and signatures in primary and early-recurrent HCC. **a** Six substitution patterns sorted by the total mutation number in primary tumors and recurrent tumors from all 40 patients (left panel), 18 patients with de novo recurrence (middle panel), and 22 patients with ancestral recurrence (right panel). **b** Major mutational signatures extracted from paired primary and recurrent HCC samples. **c** Dot plots showing the distribution of mutational signatures to each patient in the trunk clone, the trunk subclone, and primary and recurrence branch subclones from patients with ancestral recurrence, based on our classification of clonal and subclonal mutation clusters. **d** Comparison of major mutational signatures across trunk clone, trunk subclone, and primary and recurrence branch subclones in 22 patients with ancestral recurrence. Left panel: mutational signatures with elevated proportions; middle panel: mutational signatures with decreased proportions; right panel: mutational signatures with variable proportions. **e** Dot plots showing the distribution of mutational signature to each patient in the clone and subclone of the primary or recurrent tumor, respectively, from the patients with de novo recurrence, based on our classification of clonal and subclonal mutation clusters. **f** Comparison of major mutational signatures across clone and subclone in primary tumors or recurrent tumors, respectively, from patients with de novo recurrence. Left panel: mutational signatures with elevated proportions; middle panel: mutational signatures with decreased proportions; right panel: mutational signatures with maintained proportions

We analyzed the mutational spectrum in all 40 pairs of primary and early-recurrent HCCs (Supplementary Fig. 9). Besides ubiquitous C > A transversions and C > T and T > C transitions in all of the HCCs, features which are shared by other HCC cohorts,^4–6,23,24^ we identified a dominant T > A transversion pattern in all of the hypermutated tumor samples (mutation rate > 9/Mb, 5 primary and 5 recurrent), suggesting that the T > A transversion contributes to somatic SNVs in Chinese patients with HCC, which is consistent with our recent study.^9^ We did not find a significant difference in mutational spectrum between the primary HCCs and the recurrent HCCs across the entire sample or in the subgroups of de novo recurrence and ancestral recurrence (Fig. 5a).

To further determine the temporal dynamics of the primary and recurrent HCC genomic landscapes, we analyzed mutational signatures in all 40 pairs of primary and early-recurrent HCCs using DeconstructSigs, which accurately reconstructs the mutational profiles of samples on the basis of a predefined mutational spectrum of 30 COSMIC signatures. Corresponding to the mutational spectrum, signature 22, which is characterized by dominant T > A mutations and known to result from exposure to aristolochic acid (AA) and to be associated with a high mutational burden,^9,25^ was a predominant signature in all of the hypermutated HCC samples (Supplementary Fig. 9).

Mutational signature analysis showed that the first striking feature is that the mutational signatures were similar in the primary tumors and recurrent tumors, even in the patients with de novo recurrence regardless of the evolutionary stage of the tumors (Fig. 5b, e, f; Supplementary Fig. 11). That suggests that although the recurrent tumors developed genetically independently of the primary tumors, they followed a similar mutational process. A second striking feature revealed by the mutational signature analysis is that the heterogeneity in mutational signatures across patients was considerably greater than the heterogeneity across different evolutionary stages within a given tumor (Supplementary Figs. 10, 11). That suggests that a given HCC accesses only a subset of the mutational processes that are potentially available to it, but those mutational processes contribute genomic variation on an ongoing basis. Nonetheless, there were some shifts in the relative contributions of mutational processes over time. In the patients with ancestral recurrence, the relative contributions of signatures displayed relatively greater variation between trunk mutations and branch mutations, whereas there was limited variation between trunk clone and trunk subclone mutations. For example, tobacco consumption-related signature 4, AA signature 22, and aflatoxin exposure-related signature 24 displayed prominent enrichment in the trunk clonal and trunk subclonal events but were dramatically decreased in the primary or recurrent branch subclonal events (Fig. 5c, d), suggesting that those etiologies mainly contributed to HCC formation and development but not to the intra-hepatic dissemination stage. Among these patients, P51 was an interesting exception, aflatoxin exposure-related signature 24 was enriched in the trunk mutations, especially in the trunk clonal events (Supplementary Fig. 10), suggesting that the aflatoxin-related etiology contributed to tumor formation and development in that patient. AA signature 22 was specifically identified in recurrence subclonal events, which is consistent with a medical history of taking Chinese herbs containing AA after the first curative resection. We also found that signature 12 gradually decreased during the course of HCC progression, but the etiology of that signature is still unknown. In contrast, signature 3 (homologous recombination deficiency; HRD), signature 5 (aging), and signature 8 (unknown etiology) were elevated in branch subclonal events, suggesting that the greater contributions of those mutational processes occurred in the stage of HCC intra-hepatic dissemination. In addition, signatures 9 and 16 displayed variable contributions in different evolutionary stages of HCC ancestral recurrence. Signature 9 is characterized by a pattern of mutations that has been attributed to polymerase η, whereas signature 16 is related to alcohol consumption. Those two etiologies might have complicated effects during HCC ancestral recurrence.

In the patients with de novo recurrence, most signatures were stable between the clonal mutations and the subclonal mutations in both the primary tumors and the recurrent tumors, including signatures 4, 8, 12, 16, 22, and 24 (Fig. 5e, f), which suggests that the etiologies related to signatures affect the entire evolutionary process during HCC de novo recurrence. In addition, we observed that signature 5 was increased in the subclonal events, which is consistent with the pattern in ancestral recurrence, implying that the effects of aging-related mutational processes are more important in the latter stage of HCC early recurrence. Signatures 9 and 28 only occurred in the clonal mutations in a subset of patients, suggesting that the etiologies related to those signatures might have little effect on the latter evolutionary process during HCC de novo recurrence, although the etiology of signature 28 is still unknown.

### Multi-region WGS revealed tumor spatiotemporal heterogeneity and polyclonal, monophyletic dissemination in HCC ancestral recurrence

To further explore the heterogeneity and clonal progression of HCC with ancestral recurrence, we performed multi-regional WGS on four of the patients (P42, P44, P51, and P58). Together with the four pairs of primary and recurrent tumors, we sequenced a total of 15 primary tumor samples and 15 recurrent tumor samples from those four patients at the whole-genome level. We obtained an average of 65.6-fold sequencing depth for the primary tumors and 62.5-fold sequencing depth for the recurrent tumors (Supplementary Table 13). We identified an average of 15,270 somatic mutations per recurrent tumor, which was significantly more than the number of somatic mutations per primary tumor (14,633, *P* = 0.0002, paired *t* test; Supplementary Fig. 12a**;** Supplementary Table 14). We also detected a mean of 37.3 SVs (range: 6–77) per primary tumor and a mean of 33.6 SVs (range: 0–98) per recurrent tumor (Supplementary Fig. 12b; Supplementary Table 15). Tumor ploidy analysis revealed that WGD occurred in all of the tumors from patient P51 and all of the recurrent tumors from patient P44 (Supplementary Fig. 12a).

**Fig. 6:**
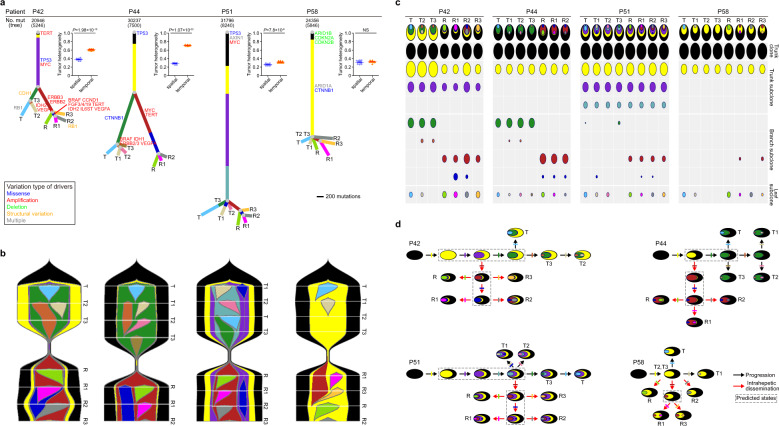
Spatiotemporal heterogeneity and polyclonal dissemination in four patients with ancestral recurrence revealed by multi-region WGS. **a** Phylogenetic trees constructed from four patients with ancestral recurrence (total of 30 tumor samples). Trees were derived from somatic mutations (SNVs + indels) in genic region based on subclonal architectures. The numbers of all genome-wide somatic mutations per patient are labeled above the tree (the numbers of somatic mutations in genic region involved in constructing the phylogenetic trees are labeled in brackets). Lengths of lines are proportional to the number of mutations in each cluster. Quantification of tumor spatial and temporal heterogeneity of each patient is on the right of the corresponding phylogenetic tree. **b** Fishplot showing the tumor clonal evolution process from primary tumor to early-recurrent tumor. **c** Oval plots showing the subclonal structure of tumor samples from each patient. The diameter of each oval is proportional to the corresponding CCF value. Subclonal structures are illustrated by the nested ovals. **d** Clonal evolutionary history. The dashed box shows the predicted historical states during the tumor ancestral recurrence. Black arrows denote clonal evolution during primary tumor progression. Red arrows denote clonal evolution from the primary tumor to recurrent tumor. Arrows are labeled the acquisition of new subclones with orthogonal lines. Each colored line corresponds to a subclone

We explored the spatial and temporal tumor heterogeneity based on somatic mutations (SNVs + indels) at the whole-genome level. We defined spatial heterogeneity as intra-tumoral heterogeneity between paired regions within primary or recurrent tumors. We defined temporal heterogeneity as inter-tumoral heterogeneity between paired regions from primary and recurrent HCCs, respectively. In three of the four patients (P42, P44, and P51), we observed substantially higher temporal heterogeneity than spatial heterogeneity (Fig. 6a), which was consistent with the Jaccard similarity coefficient results (Supplementary Fig. 12c).

We constructed phylogenetic trees, analyzed the subclonal architectures, and displayed evolutionary processes using fishplot (Fig. 6a–c; Supplementary Figs. 13–17; Supplementary Tables 16−19). We determined the clonal relationships among the constituent subclones and found evidence for polyclonal seeding of recurrence: multiple mutational clusters presented subclonally in more than one recurrent region (Fig. 6c). Hence, the recurrences were most likely seeded by multiple distinct subclones derived from the primary tumors.^26,27^ In three of the four patients (P42, P44, and P51), besides one main clone (black oval) and one to three subclones (yellow, purple, and gray blue ovals) with varying CCFs in each primary and recurrent tumor region, we identified a red cluster containing subclonal mutations in every recurrent tumor region but not in primary tumor regions, which suggests that that cluster might contain important metastasis-promoting drivers such as *ERBB2/3* amplification in patient P42 and *MYC* and *TERT* amplification in patient P44, which is also indicative monophyletic dissemination.^28^ In patient P58, although the red subclone affected only the R1 and R3 samples of the recurrent tumor, we inferred the possibility of monophyletic dissemination during early tumor recurrence. In addition, we found evidence of branching spreading patterns. In patient P51 for example, the blue inter-site subclones in R1 and R2 were descend from T1 (Fig. 6b, c). The tumor evolutionary trajectory and history during HCC progression and intra-hepatic dissemination are presented in Fig. 6d. Collectively, our results revealed spatiotemporal heterogeneity and uncovered polyclonal, monophyletic dissemination that was collectively shaped by branched spreading in HCC ancestral recurrence.

### *BCL9* was mutated and frequently copy number gained specifically in recurrent tumors during HCC early recurrence

**Fig. 7:**
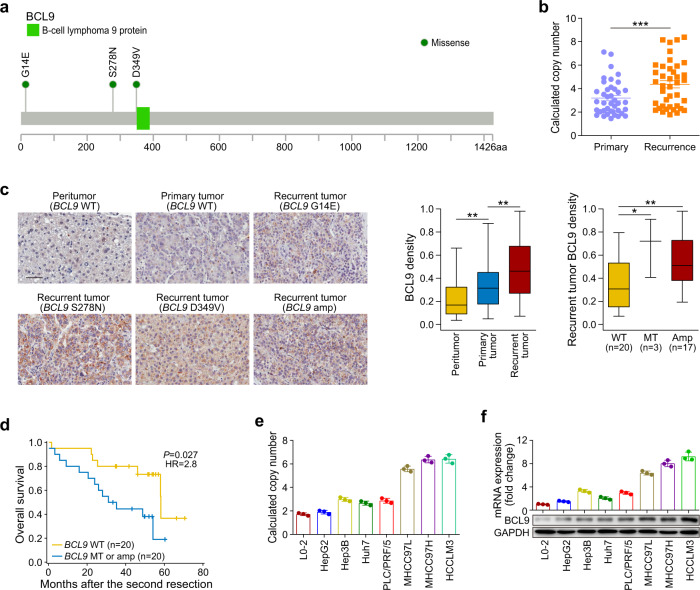
Clinical significance of BCL9 genomic alteration in 40 pairs of primary and recurrent HCCs. **a** Distribution of somatic mutations in *BCL9* identified in the recurrent HCC samples. **b** Calculated *BCL9* copy numbers identified in 40 pairs of primary and recurrent HCCs, ***P < 0.001. **c** Representative BCL9 staining and its density statistics in peritumor tissues, primary tumor tissues with wild-type *BCL9*, and recurrent tumor tissues with mutant *BCL9* (G14E, S278N, and D349V) and *BCL9* amplification; scale bars = 50 μm, *P < 0.05, **P < 0.01. **d** Kaplan−Meier survival analysis showing overall survival rates after the second resection based on *BCL9* mutation and copy-number variation in recurrent tumors. **e** *BCL9* copy numbers revealed by qPCR in one normal liver cell line (L0-2) and six HCC cell lines. **f** BCL9 mRNA and protein levels examined by qRT-PCR and western blot in one normal liver cell line and six HCC cell lines

We identified somatic mutations in *BCL9* in 3 of the 40 (7.5%) recurrent tumors overall, including 1 sample of ancestral recurrence and 2 samples of de novo recurrence, with no *BCL9* mutations in the primary tumors (Fig. 7a). *BCL9* is located within a large genomic region of chromosome 1q21 (chr1: 143657015-147924399) that was frequently gained in the recurrent tumors (Supplementary Table 4). We validated those results using Sanger sequencing and qPCR, which showed that somatic mutations and copy-number gains of *BCL9* occurred in 7.5% (3 of 40) and 65% (26 of 40), respectively, of the 40 samples of recurrent HCC tumors (Fig. 7a, b; Supplementary Fig. 18).

We further evaluated BCL9 expression by immunohistochemistry in all 40 HCCs. The results showed that BCL9 expression was up-regulated in the tumor samples, especially in the recurrent tumor samples, compared with that in adjacent non-tumor liver samples (Fig. 7c). Patients with *BCL9* somatic mutation or amplifications in their recurrent tumors showed a further increase in BCL9 expression (Fig. 7c). Kaplan−Meier survival analysis showed that the survival rates after the second curative resection of patients with *BCL9* somatic mutations or amplifications in their recurrent tumor were significantly lower than those of patients without such changes (Fig. 7d). These results suggest a possible oncogenic role for *BCL9* in HCC early recurrence.

### *BCL9* exerts an oncogenic role in HCC

To test the functional effect of *BCL9* in HCC, we analyzed the genotypes and expression of *BCL9* in seven HCC cell lines. All seven HCC cell lines were validated as wild type (WT) by Sanger sequencing, using the same filter criteria used for the WGS of the HCC samples. The qPCR results showed that besides HepG2, six of the HCC cell lines had *BCL9* copy-number gains, especially the highly metastatic HCC cell lines MHCC97L, MHCC97H, and HCCLM3, which exhibited *BCL9* amplification (Fig. 7e). Western blot and qRT-PCR results confirmed that the BCL9 expression levels in the seven established HCC cell lines, especially MHCC97L, MHCC97H, and HCCLM3, were increased in comparison with those in the non-transformed hepatic cell line L0-2 (Fig. 7f).

**Fig. 8:**
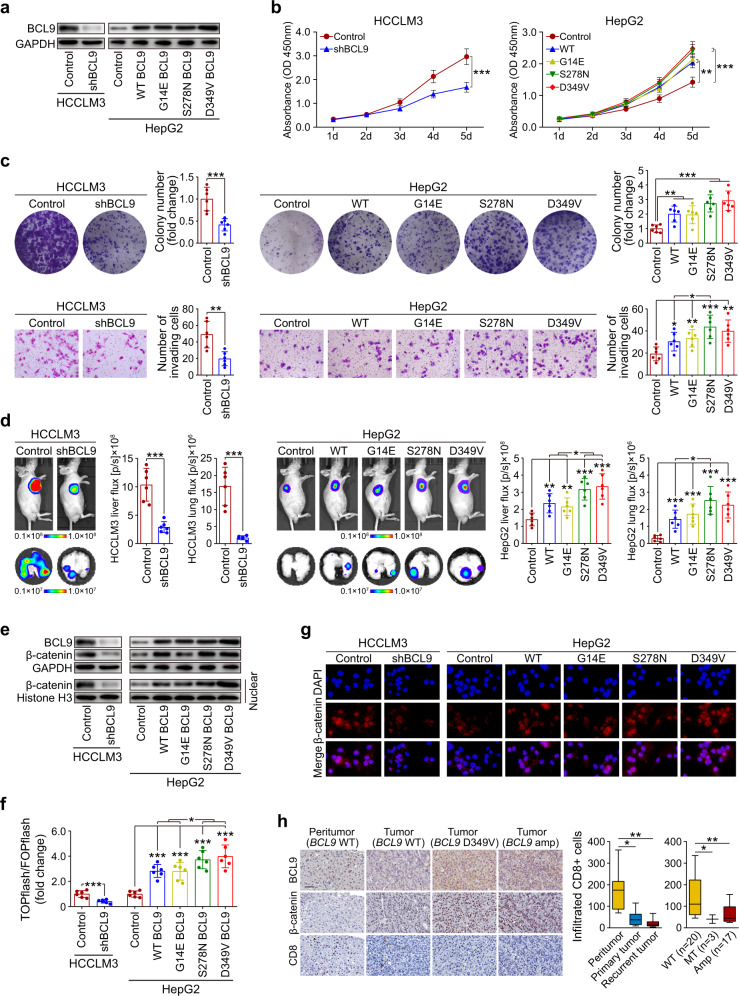
Oncogenic role of BCL9 in HCC. **a** BCL9 expression examined by western blot in stably transfected cells. **b** Proliferation of HCCLM3 cells after BCL9 knockdown and of HepG2 cells expressing wild-type or mutant BCL9 compared with that of controls, **P < 0.01, ***P < 0.001. **c** Colony formation and invasion of HCCLM3 cells after BCL9 knockdown and of HepG2 cells expressing wild-type or mutant BCL9 compared with that of controls. The bar graphs illustrate the quantification of the assay results, *P < 0.05, **P < 0.01, ***P < 0.001. **d** Representative bioluminescence images of mouse liver tumors and pulmonary metastasis. The color scale bar depicts the photon flux emitted from the mice, *P < 0.05, **P < 0.01, ***P < 0.001. **e** Western blot showed the expression of β-catenin in HCCLM3 cells after BCL9 knockdown and of HepG2 cells expressing wild-type or mutant *BCL9*. **f** Results of dual-luciferase assays; reporter activity was normalized to *Renilla* luciferase activity, *P < 0.05, ***P < 0.001. **g** Immunofluorescence staining showing subcellular β-catenin localization in the indicated cells. **h** Representative BCL9 and CD8 staining in peritumor tissues and tumor samples from patients with HCC. The color scale bar depicts the CD8-positive cell number in all 40 matched sets of peritumor, primary tumor, and recurrent tumor tissues (left panel) and in 40 recurrent tumor tissues (right panel); scale bars = 50 μm, *P < 0.05, **P < 0.01

Next, we knocked down BCL9 in HCCLM3 cells (Fig. 8a). Biofunctional investigations revealed that the knockdown of BCL9 resulted in decreases in HCC cell proliferation, colony formation, and invasive ability (Fig. 8b, c and Supplementary Fig. 19). In vivo HCC mouse models showed that BCL9 knockdown reduced tumor growth and pulmonary metastasis (Fig. 8d).

We generated lentiviral constructs to re-express WT BCL9 or three mutant BCL9 variants in HepG2 cells (Fig. 8a). The results showed that overexpression of WT or mutant BCL9 significantly enhanced HCC cell proliferation, colony formation, and invasion ability. In particular, the expression of BCL9^D349V^ and BCL9^S278N^ resulted in more prominent enhancement (Fig. 8b, c and Supplementary Fig. 19), indicating that the two types of mutations were possibly activating. In agreement with the in vitro studies, analysis of an in vivo HCC mouse model showed that the WT or mutant BCL9, especially BCL9^D349V^ and BCL9^S278N^, substantially promoted tumor growth and pulmonary metastasis (Fig. 8d). In vitro functional effect of *BCL9* on PLC/PRF/5 cells and MHCC97H cells revealed consistent results (Supplementary Fig. 20). Those results support the notion that *BCL9* exerts an oncogenic role in HCC and that certain activating mutations further enhance its tumor-promoting effect.

### *BCL9* contributes to the activation of WNT/β-catenin signaling and an immune-excluded tumor microenvironment

BCL9, known as a coactivator of β-catenin–mediated transcription, is highly expressed in several types of cancer.^29^ Hence, we tested the effect of BCL9 on WNT/β-catenin signaling in HCC cells. Western blot analysis showed that total β-catenin expression was reduced in HCCLM3 cells after knockdown of BCL9, whereas it was upregulated in HepG2 cells following BCL9 overexpression and upregulated even further following BCL9 mutation (Fig. 8e). Knockdown of BCL9 also markedly decreased the transactivating activity of β-catenin in HCCLM3 cells, as determined by β-catenin reporter assay. Conversely, overexpression of WT BCL9 induced TCF/LEF activities in HepG2 cells, and BCL9 mutation induced even higher activity levels (Fig. 8f). Furthermore, subcellular fractionation (Fig. 8e) and immunofluorescence staining (Fig. 8g) showed that knockdown of BCL9 resulted in a substantial decrease of nuclear β-catenin in HCCLM3 cells, whereas overexpression of BCL9, especially mutant BCL9, led to nuclear accumulation of β-catenin in HepG2 cells. We observed similar results in the 40 pairs of primary and recurrent HCC samples, which showed nuclear accumulation of β-catenin in samples with BCL9 mutation or amplification, accompanied by decreased CD8+ cell infiltration (Fig. 8h). These results suggest that BCL9 contributes to the activation of WNT/β-catenin signaling in HCC cells and an immune-excluded tumor microenvironment.

## Discussion

Tumor evolution is a dynamic process both spatially and temporally. A better understanding of that evolutionary process would provide clues to guide effective therapies. Using whole-genome analysis of matched primary and early-recurrent tumors from patients with HCC, we obtained findings that have immediate clinical and biological implications. First, we identified two patterns of HCC early recurrence (ancestral recurrence and de novo recurrence) based on the proportion of shared somatic mutations and clonality indices between primary and early-recurrent tumors. We further confirmed those two patterns through the following two findings: (1) shared mutations in patients with de novo recurrence were nearly all subclones and included no driver mutations; (2) driver alterations between primary and recurrent tumors in a given patient with de novo recurrence were entirely different.

A large degree of intra-tumor heterogeneity (ITH) has been found in several types of cancer.^30^ Researchers at our institute recently confirmed that the mean percentage of ITH in HCC is 39% (range: 12.9–68.5%).^31^ We also revealed various degrees of ITH in paired multi-regional primary—early recurrent tumors from four of those patients with HCC (23–42%; Fig. 6a). That degree of ITH was far lower than the heterogeneity between the primary tumors and the de novo recurrent tumors in the present study (mean = 97.4%, range: 94.7–99.4%). Those data suggest that although ITH exists in HCC, it cannot account for the extremely high heterogeneity between primary tumors and de novo recurrent tumors.

Previous delineation of HCC recurrence patterns and potential mechanisms were based on recurrence time. Early recurrence, occurring within 2 years of initial therapy, was attributed either to micrometastases within the liver but outside the treated area or to incomplete initial treatment. Late recurrence, on the other hand, was attributed to new cancers.^16^ Our results show, however, that even in early HCC recurrence, about 45% (18 of 40) of patients experienced de novo recurrence actually caused by a second primary lesion. Furthermore, the recurrence pattern was not associated with the time of recurrence but was correlated with the recurrence location: distant recurrence was prone to be de novo, whereas while local recurrence tended to be ancestral. In addition, recurrent tumors of ancestral origin tended to be associated with more hallmarks of progression than de novo recurrent tumors, such as WGD, large tumor size, and poor survival time. We also observed that liver cirrhosis tended to display the de novo pattern, whereas no liver cirrhosis tended to display the ancestral pattern, either at the time of the first resection (*P* = 0.053) or the second resection (*P* = 0.119); however, there was no statistical significance, possibly due to the limited sample size (Supplementary Fig. 21). Therefore, de novo recurrence may be partly explained by an oncogenic field effect in cirrhotic livers.

The high rate of early recurrence in HCC limits long-term patient survival after surgical resection. The ability to distinguish between different recurrence patterns might have a great impact on the scientific formulation of individualized therapies and evaluations of clinical outcomes. In principle, ancestral recurrence is attributable to micrometastases that occur in the liver outside the treated area after the first resection. Therefore, ancestral recurrence is more suitable for comprehensive treatment, including targeted therapy and interventions such as radiofrequency ablation and transcatheter hepatic arterial chemoembolization. De novo recurrence is essentially a de novo primary tumor and therefore is naturally more suitable for surgical resection or liver transplantation, which are expected to yield the same clinical outcome as the first resection for primary HCC.^32^

Tumor metastasis is a dynamic, multi-step process both spatially and temporally. In order to clarify the evolutionary trajectory of HCC ancestral recurrence, the result of intra-hepatic metastatic seeding, we performed deep multi-region WGS of primary tumors and recurrent tumors, which can provide accurate information about the multi-sample subclonal architecture.^18,26,28^ We revealed extensive tumoral spatial and temporal heterogeneity. More importantly, we demonstrated clear evidence of polyclonal metastatic seeding, which suggests that metastatic lesions result either from multiple waves of migrating cells or, alternatively, from simultaneously migrating clusters of cells composed of genetically distinct clones.^28^ Our analysis also revealed a monophyletic dissemination model for polyclonal metastatic seeding in HCC ancestral recurrence. In that model, metastatic potential is acquired once in the evolution of the primary tumor, which ensures that the capability to metastasize is inherited by one subclone that subsequently arise. Thus, polyclonal dissemination might have a monophyletic origin such that all metastatic clones share a recent common ancestor.^28^ Although Kan’s study^8^ delineated recurrent mutations in HCC through WGS, it only contained primary tumor samples. Ding et al also investigated the genomic and epigenomic features of primary and recurrent HCCs; however, only 9 patients were analyzed by WGS and 6 patients were analyzed by whole-exome sequencing. The limited sample size may impede the accuracy of classification of recurrence patterns and cannot infer the general evolutionary trajectory during HCC early recurrence.

By comparing genomic alterations between primary tumors and recurrent tumors, we identified a gene that was specifically mutated and frequently copy number gained in recurrent tumors, *BCL9*. *BCL9* was mutated in 0.4–2.2% of primary HCC samples in other studies from Japan, South Korea, Europe, and the TCGA cohort,^2–7^ which also suggested that alterations of *BCL9* are especially common in recurrent HCC tumors. BCL9 plays an essential role as a coactivator in the WNT/β-catenin signaling pathway by mediating the recruitment of pygopus to the nuclear β-catenin-TCF complex.^29^ BCL9 is frequently overexpressed in a variety of solid tumors including colorectal cancer, multiple myeloma, and HCC.^33^ We revealed through gain-of-function and loss-of-function studies that BCL9 plays an oncogenic role in HCC: its activation contributes to WNT/β-catenin signaling hyperactivation, an immune-excluded tumor microenvironment, and tumor growth and metastasis. This result was consistent with previous findings,^34^ which might contribute to innate resistance to anti–PD-1/PD-L1 or similar immune-checkpoint therapies. Thus, we presumed that in BCL9-mutated or amplified recurrent HCC (around 50% of recurrent tumor), combining targeting BCL9 and immune-checkpoint therapies may act as a novel therapeutic approach.

**Fig. 9:**
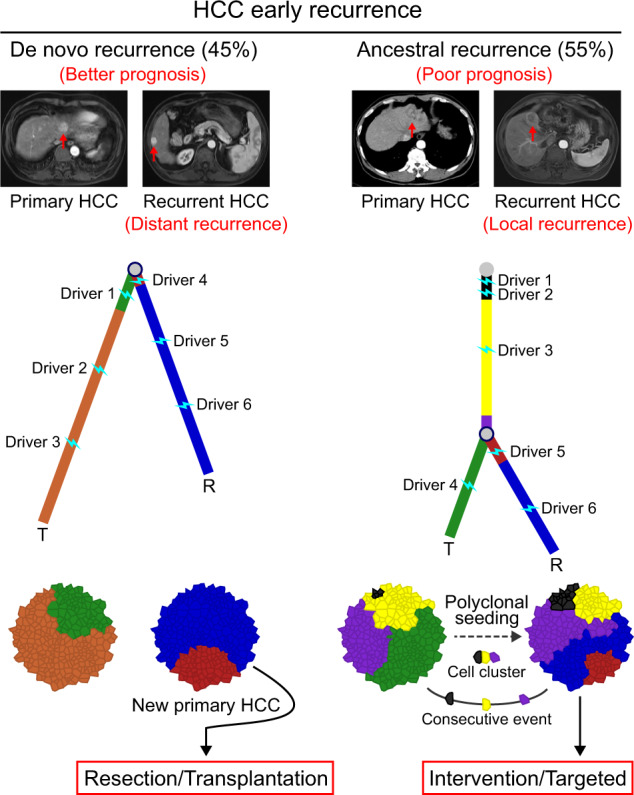
Schematic figure illustrating the recurrence pattern and evolutionary trajectory of HBV-related hepatocellular carcinoma early recurrence

In summary, using WGS, we presented a landscape of the genomic evolution during HCC early recurrence with unprecedented clarity (Fig. 9), which provides an important molecular foundation for an enhanced understanding of HCC and has implications for personalized therapy to improve patient survival.

## Materials and methods

### Patients and sample collection

Paired primary tumor, recurrent tumor, and normal liver tissues were obtained from surgical resection specimens from patients with HCC at the Liver Surgery Department, Zhongshan Hospital, Fudan University. The Research Ethics Committee of Zhongshan Hospital approved the ethical use of human subjects for this study, and informed consent was obtained from each patient. The criteria for HCC patient selection for WGS are shown in Supplementary Fig. 1. The median time from first curative resection to recurrence in the 40 HCC patients included in the WGS analysis was 13.4 months (range: 3–23.9 months; Supplementary Table 1).

The histopathological diagnosis was based on the World Health Organization criteria. The tumor grade was determined in accordance with the classification proposed by Edmondson and Steiner.^35^ The Child-Pugh scoring system was used to assess liver function. The tumor stage was determined according to the tumor-node-metastasis classification system established by the 2017 International Union Against Cancer.^36^ Postsurgical patient surveillance was performed as previously described.^37^ Overall survival (OS) was defined as the interval between surgery and death or between surgery and the last observation point. For surviving patients, the data were censored at the last follow-up.

The human liver is a solid spatial organ that has regenerative ability after hepatic resection. Furthermore, in our study of 40 HCCs, most of the HCCs were HBV-related and presented liver cirrhosis, making it hard to calculate the exact distance between the primary and recurrent tumors. To roughly estimate the recurrence location, we defined the interval between hepatic segments nearest to the primary and recurrent tumors, respectively, as the tumor hepatic segment interval (HSI). For example, if the primary and recurrent tumors both located in hepatic segment VI, or the primary tumor was located in hepatic segments V and VI and the recurrent tumor was located in hepatic segments VI and VII, the tumor HSI was defined as 0. If the primary and recurrent tumors were located in hepatic segments VI and VII, respectively, or the primary tumor was located in hepatic segments V and VI and the recurrent tumor was located in hepatic segments VII and VIII, the tumor HSI was defined as 1, and so on. Hence, the range of tumor HSI was 0–4 (Supplementary Table 7). If the paired primary/recurrent tumors carried tumor HSI 0 or 1, we defined the recurrence as local recurrence. Otherwise, we classified the recurrence as distant (HSI: 2–4).

### DNA extraction and whole-genome sequencing

Snap-frozen tissue samples from tumors and matched non-cancerous liver were obtained and embedded in OCT compound, sectioned by a cryostat, and stained with hematoxylin and eosin. We performed macrodissection to enrich the tumor fraction relative to the dominant stromal component and other normal cells. DNA was extracted using a general protocol for genome sequencing. Preparation of sequencing libraries and DNA capture methods were carried out according to the manufacturer’s protocols. Genomic DNA was randomly broken into manageable fragments to facilitate construction of insert libraries. For human genome re-sequencing, paired-end libraries with a 400–500 bp span size were used. The fragments of template DNA from the constructed libraries were hybridized to the cell surface and then subjected to amplification to form clusters. The DNA fragments were then sequenced using an Illumina HiSeq X sequencing system. A paired-end read length of 150 bp was used in high-throughput WGS.

### Date quality control, reads mapping, and contamination calculation

Sequence artifacts, including reads containing adapter contamination, low-quality nucleotides, and unrecognizable nucleotides (N), undoubtedly set a barrier to subsequent reliable bioinformatics analysis. Hence, quality control is an essential step and is applied to guarantee a meaningful downstream analysis. The steps of data processing were as follows:

1. Discard a pair of reads if either read contains adapter contamination;
2. Discard a pair of reads if it contains poly-N;
3. Discard a pair of reads if the proportion of low-quality (Phred quality<5) bases is over 50% in either read.

Valid sequencing data were mapped to the reference human genome (UCSC hg19) using the Burrows−Wheeler Aligner (BWA)^38^ software to get the original mapping results stored in BAM format. We performed local realignment of the original BAM alignment using GATK2^39^ and then marked duplicate reads using Sambamba.^40^ We used the Conpair program^41^ to estimate the sample cross-individual contamination levels. A total of 40 normal-primary-recurrence matched samples from patients with HCC with contamination less than 2% (maximum 1.03%, minimum 0.01%) were included in the downstream analysis.

### Detection of somatic genetic alterations

Somatic SNVs were detected using muTect,^42^ and somatic indels were detected using Strelka.^43^ High-confidence somatic mutations were called if the following criteria were met: (1) both the tumor samples and the normal samples were covered sufficiently (≥10×) at the genomic level; (2) the variants were supported by at least 5% of the total reads in the tumor and less than 1% of the total reads in the normal tissue; (3) the variants were supported by at least three reads in the tumor. ANNOVAR^44^ was performed for annotation in the Variant Call Format obtained in the previous effort. Somatic SNVs and indels that were referenced in the 1000 Genomes Project with a minor allele frequency over 1% or that were located in segmental duplications were removed along with common SNPs.

To precisely determine the presence or absence of mutations in distinct samples from the same patient, we used additional two-step methods called “CNV drop” and “Force calling” to detect SNVs and indels in each sample on the basis of the aggregate set of somatic events in the patient.^18,45^ CNV drop is related to region-specific somatic mutations. If a mutation was identified in one sample but was located in a CNV loss region (cnv ≤ 1) in any other samples from the same patient that did not carry the mutation, such somatic mutations were excluded. “Force calling” is related to allele frequency-specific somatic mutations. Some mutations might be present in multiple samples but only called in a subset of samples because of low allele frequencies in the other sample(s). Such somatic mutations were recalled if they were covered sufficiently (≥10×) and supported by at least two reads.

### Tumor copy number variation detection, ploidy and purity prediction, and whole-genome duplication (WGD) status determination

We used FACETS,^46^ an allele-specific copy number analysis tool to detect genome-wide total, allele-specific, and integer DNA copy numbers and to predict tumor ploidy and purity. The GISTIC (V2.0) algorithm was used to infer recurrently amplified or deleted genomic regions.^47^ Genes with a total copy number greater than the gene-level median ploidy were considered gains. Genes with more than twice the median ploidy were considered amplifications. Genes with less than the median ploidy were considered losses. Genes with a total copy number of 0 were considered deletions. Tumors were considered to have undergone WGD if their ploidy was greater than 2.9, considering that the median ploidy of tumors that underwent WGD was 3.3 [interquartile range (IQR): 2.9–3.8] in the pan cancer study.^20^

### Detection of somatic structural variants and HBV/AAV2 integration

To detect somatic breakpoints, we used the MeerKat^48^ software with default parameters. Firstly, it predicts SVs from discordant read pairs. Secondly, it looks for reads that cover the predicted breakpoints junctions (split read support), refines breakpoints by local alignments, and predicts mechanisms that SVs are formed. High-confidence somatic breakpoints were called if the following criteria were met: (1) the breakpoint was supported by ≥10 read pairs or reads (discordant read pairs + split reads); (2) the breakpoint was supported by ≥5 discordant read pairs; (3) the breakpoint was supported by ≥3 split reads; and (4) the breakpoint was not located in segmental duplications. Two precise classification is referred as following, deletions with insertion: deletion with insertion at the break point, insertion comes from the same or a different chromosome; deletion with inversion: deletion with inversion at the break point, inversion comes from deleted part.

To infer HBV/AAV2 genotypes and identify integration sites, valid sequencing data were mapped to the human genome (NCBI build 37, hg19), HBV genomes (AB014381, AB032431, AB033554, AB036910, AB064310, AF100309, AF160501, AF223965, AF405706, AY090454, AY090457, AY090460, AY123041, D00329, M32138, NC_003977, X02763, X04615, X51970, X65259, X69798, X75657, and X85254), and the AAV2 genome (AF043303.1).^49^ Breakpoints with >3 chimeric reads aligned on both the human genome and the viral genomes were further analyzed and annotated. As integrated HBV/AAV2 was considered a strong cis-activator that can influence flanking genes over long distances (up to 1 Mb for upstream enhancers and 850 kb for downstream enhancers),^50^ samples with breakpoints within 500 kb of annotated genes were considered to be affected by HBV-integration/AAV2-integration events.

### Clonality analysis

To assess whether the primary and recurrent tumors of a given patient were clonally related, we used the clonality index (CI) to quantitate the likelihood of two tumors sharing mutations that were not expected to have co-occurred by chance.^19^

Given the repertoire of mutations in two samples, the probability of observing a given mutation in both samples is given by the binomial probability $P(X) = C_n^kp^k(1 - p)^{n - k}$, *n* = 2, *k* = 2, with p defined as, for mutations previously observed in the TCGA HCC cohort (*n* = 377),^3^ the number of occurrences of the specific mutation divided by the total number of mutations found in the cohort. Thus, the probability of observing a given set of *M* identical mutations in the two samples is given by $\mathop {\prod }\limits_{m = 1}^M P(X)_m$. The CI is defined as ${\rm{CI}} = - {\rm{log}}_{10}\mathop {\prod }\limits_{m = 1}^M P(X)_m$ and was computed separately for the 40 pairs of primary and recurrent tumors on the basis of non-synonymous SNVs and indels.

To objectively define a cut-off for clonal relatedness, we used the mutational data from the 200 unrelated HCCs from the TCGA. As the positive control (i.e., clonally related), we randomly selected 40, 60, and 80% of the set of mutations from the 200 unrelated HCCs in duplicate to simulate heterogeneity between biologically related samples. As the negative control (i.e., unrelated), we randomly selected an equivalent number of pairs (i.e., 3 × 200 = 600) of unrelated HCCs from the TCGA.

To define the optimum cutoffs, the R package ‘ROCR’ was used to maximize accuracy. To avoid over-fitting the data, the above procedures were repeated 100 times to define the median of the optimum cut-off. In this study, the median cut-off was 36.59.

### Cancer cell fraction estimation and mutation cluster analysis

For each somatic mutation, the VAF was calculated using the number of reads supporting the variant allele (Rmut) and the number of reads supporting the reference allele [Rnorm; namely, VAF = Rmut/(Rmut+Rnorm)]. Then, we calculated the value of the cancer cell fractions (CCF) as follows: VAF = p*CCF/(CPNnorm (1−*p*)+p*CPNmut), where CPNmut indicates the local copy number in the tumor, CPNnorm indicates the local copy number in the normal controls (usually assumed to be 2 except for sex chromosomes), and *p* indicates the tumor purity in each sequenced sample. The VAF was defined as the VAF of each somatic mutation. CCF is represented as a distribution between 0 and 1. Then all somatic mutations (SNV and indels) in the whole genome were applied to infer the mutation cluster according to PyClone (a Bayesian clustering method). The clusters containing less than 5/1,000 of all mutations in each patient or only involving silent mutations were filtered out from further analysis, unless otherwise specified.

### Classification of clonal and subclonal mutation clusters

All filtered mutation clusters were divided into different categories in each patient in three groups: ancestral recurrence (*n* = 22), de novo recurrence (*n* = 18), and multi-regional ancestral recurrence (*n* = 4), based on shared/private in all tumors and the corresponding CCF, the precise classification is referred as following:

In the ancestral recurrence group, four categories were divided: (1) trunk clone, representing mutation cluster present in both primary and recurrent tumors in each patient with both CCF > 0.8; (2) trunk subclones, representing other mutation clusters present in both tumors in each patient; (3 and 4) branch subclones, representing mutation clusters specifically in the primary tumor or the recurrent tumor in each patient.

In the de novo recurrence group, four categories were divided: (1) primary clone, representing mutation cluster present in primary tumors with CCF > 0.8; (2) primary subclones, representing other mutation clusters present in primary tumors; (3) recurrence clone, representing mutation cluster present in recurrent tumors with CCF > 0.8; (4) recurrence subclones, representing other mutation clusters present in recurrent tumors.

In the multi-regional ancestral recurrence group, four categories were divided: (1) trunk clone, representing mutation cluster present in all tumors in each patient with all CCF > 0.8; (2) trunk subclones, representing other mutation clusters present in all tumors in each patient; (3) branch subclones, representing mutation clusters that were shared by multiple tumors in each patient; (4) leaf subclones, representing mutation clusters observed in only one tumor in each patient.

### Construction of subclonal architectures

To construct the subclonal structure of each patient, the relationships among all clones and subclones from all tumors in a given patient were determined jointly based on the pigeonhole principle.^51^ For any two subclones, the one with the smaller CCF could be either a descendant (linear relationship) or a brother/sister (branching relationship) of the subclone with the larger CCF. To determine the linear or branching relationships, we used the following rules: (1) two subclones were linear if one subclone contained mutations with larger CCFs than the other subclone in all tumors, or if the sum of the CCFs in the two subclones was greater than 1; (2) two subclones were branching if the relative CCFs in the two subclones were reversed in some tumors; (3) two branching subclones could not be both linear with an ancestral subclone if the sum of the CCFs of the branching subclones was greater than the CCF of the ancestral subclone. Nested ovals were utilized to show the subclonal architectures of each tumor. The Fishplot package was utilized to show the tumor clonal evolution in each patient.

### Phylogenetic tree construction and driver labeling

All somatic mutations (SNVs/Indels) based on subclonal architectures were used to construct the phylogenetic tree of the tumors in each patient. To calculate the relative molecular time of divergence, the relative contributions of different categories during evolution were estimated by comparison to the mutations in the primary tumor in each patient. To label the drivers to the phylogenetic trees, each coding variant was manually curated with likely driver status following a systematic approach. First, we summarized a list of potential HCC driver genes either from published reference materials consisting of the Cancer Gene Census^52^ or from a literature review of HCC sequencing studies;^2–6,9^ Next, we evaluated the types of variants in the potential HCC driver genes and identified the variants as drivers when they met one of the three following criteria: (1) non-synonymous SNVs and indels; (2) CNVs that amplified oncogenes or deleted TSGs; (3) structural variations (SVs) with any breakpoint at potential HCC driver genes.

### Spectrum and signatures of somatic mutations

Based on the classification of clonal and subclonal mutation clusters, we used deconstructSigs, a multiple regression approach, to extract mutational signatures and statistically quantify the contribution of each signature to each mutation cluster category. Student’s *t*-test was used to compare the mutational spectra of the six mutation types (C > A, C > G, C > T, T > A, T > C, and T > G) and the mutational signatures between the four categories in the ancestral recurrence group and in the de novo recurrence group.

### Sanger sequencing

We randomly selected 1,500 protein-coding SNVs and 60 indels identified by WGS. We used Sanger sequencing to validate the selected mutations. All mutations presented in Figs. 1e, 7a were also validated by Sanger sequencing. Sanger sequencing primers were designed using the Primer3 software (http://frodo.wi.mit.edu/). All mutations identified in tumors were confirmed by independent PCR and Sanger sequencing in the specific tumors and paired normal tissues to determine their somatic nature.

### Statistical analysis

Statistical analyses were performed in the R environment or using SPSS 16.0 for Windows. The data were expressed as the mean ± SD of three independent experiments unless otherwise specified. Student’s *t*-test was used to compare quantitative data between groups. The chi-square or Fisher’s exact test was used to compare categorical data. The Kaplan−Meier method was used to calculate both the OS and the cumulative recurrence rates. Differences were analyzed by the log-rank test. *P*-values <0.05 were considered statistically significant.

Other detailed information about cell lines and animals, lentiviral vectors and cell transfection, luciferase reporter assay, cell proliferation, colony formation, and matrigel invasion assays, in vivo assays for tumor growth and metastasis, RNA isolation and qRT-PCR, western blot and immunofluorescence assay, immunohistochemistry and evaluation of immunohistochemical variables can be found in the online Supplementary Materials and Methods.

## Supplementary information

Supplementary Materials

Supplementary Tables

## Data Availability

All data can be viewed in NODE (http://www.biosino.org/node) by pasting the accession OEP001774 into the text search box or through the URL: http://www.biosino.org/node/project/detail/ OEP001774.
