# Supplementary material for: Whole-genome sequencing reveals the evolutionary trajectory of HBV-related hepatocellular carcinoma early recurrence
Source: Signal Transduct Target Ther. 2022 Jan 26;7:24. doi: 10.1038/s41392-021-00838-3 (PMC8789859; doi:10.1038/s41392-021-00838-3)
Supplement: Supplementary file 1 — Supplementary Materials [file 41392_2021_838_MOESM1_ESM.docx]

Supplementary Materials for

**Whole-Genome Sequencing Reveals the Evolutionary Trajectory of HBV-Related Hepatocellular Carcinoma Early Recurrence**

Shao-Lai Zhou^1,3†^*, Zheng-Jun Zhou^1,3†^, Cheng-Li Song^4,5†^, Hao-Yang Xin^1,3†^, Zhi-Qiang Hu^1,3^, Chu-Bin Luo^1,3^, Yi-Jie Luo^4^, Jia Li^1,3^, Zhi Dai^1,3^, Xin-Rong Yang^1,3^, Ying-Hong Shi^1,3^, Zheng Wang^1,3^, Xiao-Wu Huang^1,3^, Jia Fan^1,2,3^, Jian Zhou^1,2,3^*

Correspondence to: [zhou.jian@zs-hospital.sh.cn](mailto:zhou.jian@zs-hospital.sh.cn) (JZ) or [zhoushaolai99@sina.com](mailto:zhoushaolai99@sina.com) (SLZ)

**Materials and Methods**

**Cell lines and animals**

MHCC97L, MHCC97H, and HCCLM3 are HCC cell lines established at our institute with stepwise increases in lung metastatic potential on the same genetic background.[^1^](#_ENREF_1) The normal liver cell line L-02 and the HCC cell lines HepG2, Hep3B, PLC/PRF/5 (low-metastatic human HCC cell lines, American Type Culture Collection), and Huh7 were purchased (Institute of Biochemistry and Cell Biology, Chinese Academy of Sciences, Shanghai, China). All the cell lines were identified by short tandem repeat typing. A mycoplasma detection kit (Shanghai GeneChem Co) was used to exclude mycoplasma contamination. The Ubi-MCS-Luc-IRES-puromycin lentiviral vectors were used to transfect the cell lines. Four-to-six-week-old male BALB/c nu/nu mice were obtained from the Shanghai Institute of Material Medicine (Chinese Academy of Science) and maintained under specific pathogen-free conditions. Humane care was provided for all animals in accordance with the criteria described in the ["Guide for the Care and Use of Laboratory Animals"](http://oacu.od.nih.gov/ac_cbt/guide3.htm.htm) (National Institutes of Health publication 86–23, revised 1985).

**Lentiviral vectors and cell transfection**

The following lentiviral vectors were purchased from Shanghai GeneChem Co: wild-type and mutant BCL9 expression vectors and corresponding control lentiviral vector (Ubi-MCS-3FLAG-SV40-puromycin) and the shRNA-BCL9 vector and its negative control (mU6-MCS-Ubi-Luc). The Ubi-MCS-3FLAG-SV40-puromycin-BCL9 wild-type and mutant vectors were transfected into HepG2 cells with low intrinsic BCL9 levels. The mU6-MCS-Ubi-Luc-shRNA-BCL9 vector was transfected into HCCLM3 cells with high intrinsic BCL9 expression. The Ubi-MCS-3FLAG-SV40-puromycin and mU6-MCS-Ubi-Luc lentiviral vectors were used as controls. Stably transfected clones were validated by immunoblotting. Reporter plasmids containing wild-type (CCTTTGATC; TOPflash) or mutated (CCTTTGGCC; FOPflash) TCF/LEF DNA binding sites were also purchased.

**Luciferase reporter assay**

Cells were seeded in triplicate on 24-well plates and allowed to settle for 24 h. The indicated plasmids plus 1 ng pRL-TK *Renilla* plasmid were transfected into the cells using Lipofectamine 2000 Reagent (Life Technologies). Forty-eight hours after transfection, the Dual-Luciferase Reporter Assay (Promega) was performed according to the manufacturer’s instructions, as previously described.[^2^](#_ENREF_2)

**Cell proliferation, colony formation, and Matrigel invasion assays**

Cells (2000 cells/well) were seeded in 100 μL media on a 96-well plate. Then, 10 μL CCK-8 solution (Dojindo) was added to the cells at the indicated time points. The cells were then incubated for an additional 2 h. The numbers of viable cells were determined by measurement of absorbance at 450 nm.

To assess the colony formation abilities of the cells, 500–1,000 cells were seeded into each well of six-well plates and incubated at 37°C for 12–16 days. Then, the cells were fixed with 100% methanol and stained with 0.1% crystal violet. Image-Pro Plus v6.2 (Media Cybernetics) was used to count the megascopic cell colonies.

To assay cell invasion, 24-well Transwell plates with an 8 μm pore size (Minipore) were precoated with Matrigel (BD Biosciences). Then, 100 μL Dulbecco’s modified Eagle medium (DMEM) with 1% fetal bovine serum (FBS) containing 1×10^5^ cells was added to the upper chamber. The lower chamber contained 600 μL DMEM with 10% FBS. After 48 h, both the Matrigel and the remaining cells in the upper chamber were removed. Cells that had invaded the lower surface of the membrane were fixed using 4% paraformaldehyde and then stained with Giemsa. Cells from five microscopic (200×) fields were counted.

**In vivo assays for tumor growth and metastasis**

100 μL serum-free DMEM and Matrigel (BD Biosciences; 1:1) containing 5×10^6^ HCC cells were injected subcutaneously into the upper left flank region of nude mice. The mice were monitored every 5 days and were sacrificed after 5 weeks. Bioluminescence imaging was performed using an IVIS Lumina K Series III, and image radiance values were normalized using Living Image (Perkinelmer). Upon sacrifice, the tumors were recovered, and the volume of each tumor was determined. The lungs were removed and embedded in paraffin, and the total number of lung metastases was counted under a microscope as described previously.[^3^](#_ENREF_3)

**RNA isolation and qRT-PCR**

Total RNA was extracted from cells using Trizol reagent (Invitrogen) according to the manufacturer's instructions. The mRNA expression in the cells was assessed with qRT-PCR using an ABI7900HT instrument (Applied Biosystems). Quantitative RT-PCR (qRT-PCR) was performed using the SYBR PrimeScript RT-PCR Kit (Takara Bio). GAPDH was used as an internal control for qRT-PCR. Relative mRNA levels were estimated on the basis of Ct values and normalized according to GAPDH expression using the following equation: 2^-ΔCt^(ΔCt=Ct [target gene]-Ct [GAPDH]). All experiments were performed in triplicate.

**Western blot and immunofluorescence assay**

Western blotting was performed as described previously.[^4^](#_ENREF_4) Briefly, proteins from total cell lysates were separated by 10% SDS-PAGE. The proteins were then transferred to polyvinylidene difluoride (PVDF) membranes. After washing and blocking steps, the membranes were incubated with primary antibodies. After washing, the membranes were incubated with horseradish peroxidase-conjugated secondary antibodies. Antibody binding was detected using enhanced chemiluminescence assays.

For immunofluorescence assays, cells cultured on glass slides were fixed in 4% paraformaldehyde for 15 min. Subsequently, the cells were permeabilized with 0.1% Triton X-100 for 15 min at room temperature, washed with phosphate buffered saline (PBS), and blocked with PBS containing 1% (w/v) bovine serum albumin (BSA) and 0.15% (w/v) glycine (BSA buffer) for 1 h at room temperature. The cells were then treated with primary antibody for 2 h at room temperature. A negative control (primary antibody omitted) was included on every slide. The cells were then washed with BSA buffer and incubated with 2 μg/mL Alexa Fluor 488-conjugated goat anti-mouse antibody (Molecular Probes, Eugene, OR) for 1 h at room temperature. After rinsing in PBS, the slices were counter-stained with diamidino phenylindole and examined by fluorescence microscopy (Leica Microsystems Imaging Solutions, Cambridge, UK).

**Immunohistochemistry and evaluation of immunohistochemical variables**

Immunohistochemistry staining was performed using an avidin-biotin-peroxidase complex as described previously.[^5^](#_ENREF_5) Briefly, rehydration and microwave antigen retrieval were performed. Then, the slides were incubated with monoclonal antibodies at 4°C overnight. Next, the slides were incubated with secondary antibody (GK500705, Gene Tech) at 37°C for 30 min. The slides were stained with 3,3′-diaminobenzidine and then counter-stained with Mayer’s hematoxylin. Slides that were treated identically but without primary antibody served as negative controls.

Immunohistochemistry staining was analyzed by three independent investigators who were blinded to the patient characteristics. Any discrepancies were resolved by consensus. Three representative microscope fields were photographed under high-power magnification (200×) using the Leica QWin Plus v3 software. Each image was captured using identical settings. Image-Pro Plus v6.2 software (Media Cybernetics, Inc.) was used to determine the optical density. In each photograph, the integrated optical density of positive BCL9 staining was determined. The ratio of that density to the total area of each photograph was calculated as the density. The density of CD8 staining was evaluated by quantifying the positive cells in five regions (1 mm^2^) that were randomly selected during the automated imaging analysis. The average total number of CD8-positive cells across the five areas was expressed as a cell density per mm^2^.


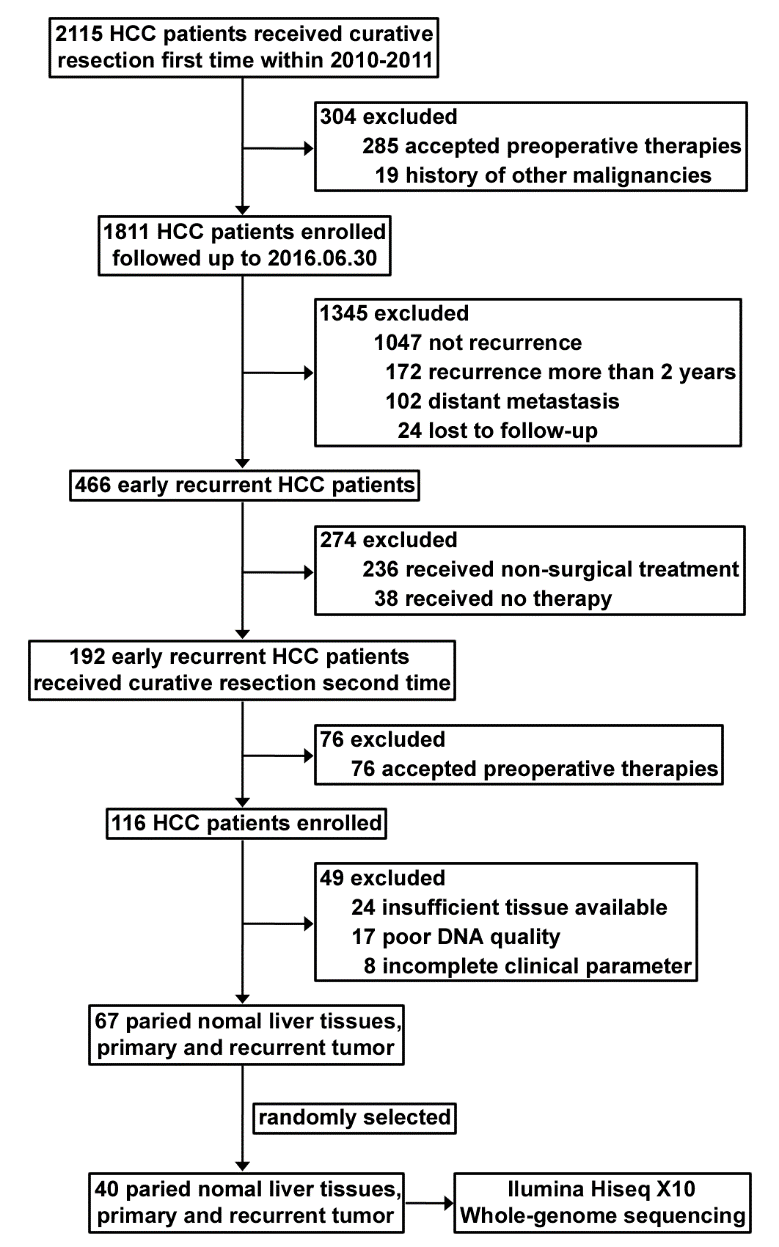


**Supplementary Fig. 1** The criteria of patient selection for WGS in this study.


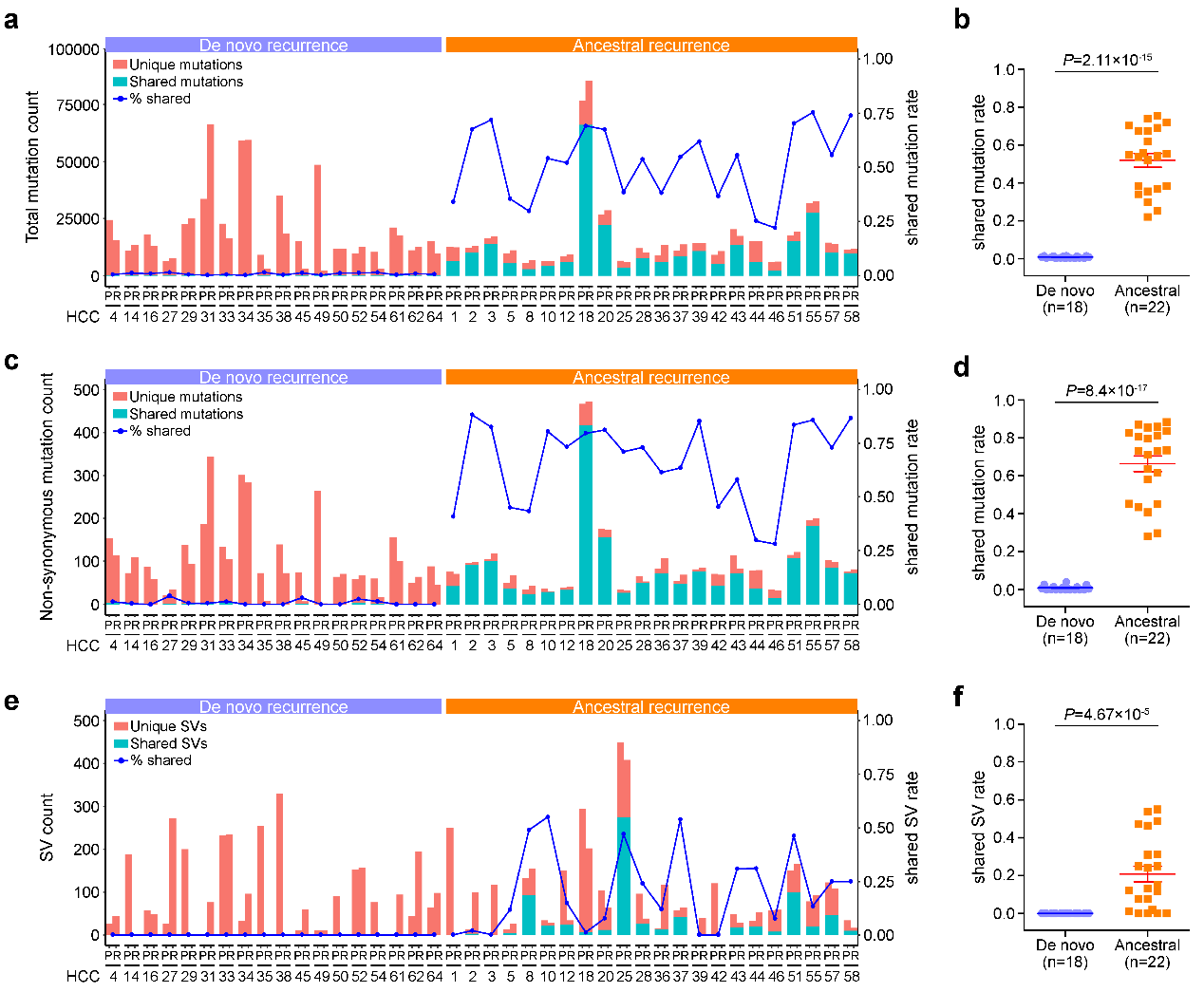


**Supplementary Fig. 2 Recurrence patterns during HCC early recurrence after curative resection. a-b** Numbers of somatic mutations (SNVs+Indels) and frequency of shared mutations at the whole-genome level between primary tumors and recurrent tumors across 40 patients with HCC after original calling. **c-d** Numbers of non-synonymous mutations and frequencies of shared mutations between primary tumors and recurrent tumors across the 40 patients after force calling. **e-f** Numbers of SVs and frequencies of shared SVs between primary tumors and recurrent tumors across the 40 patients.


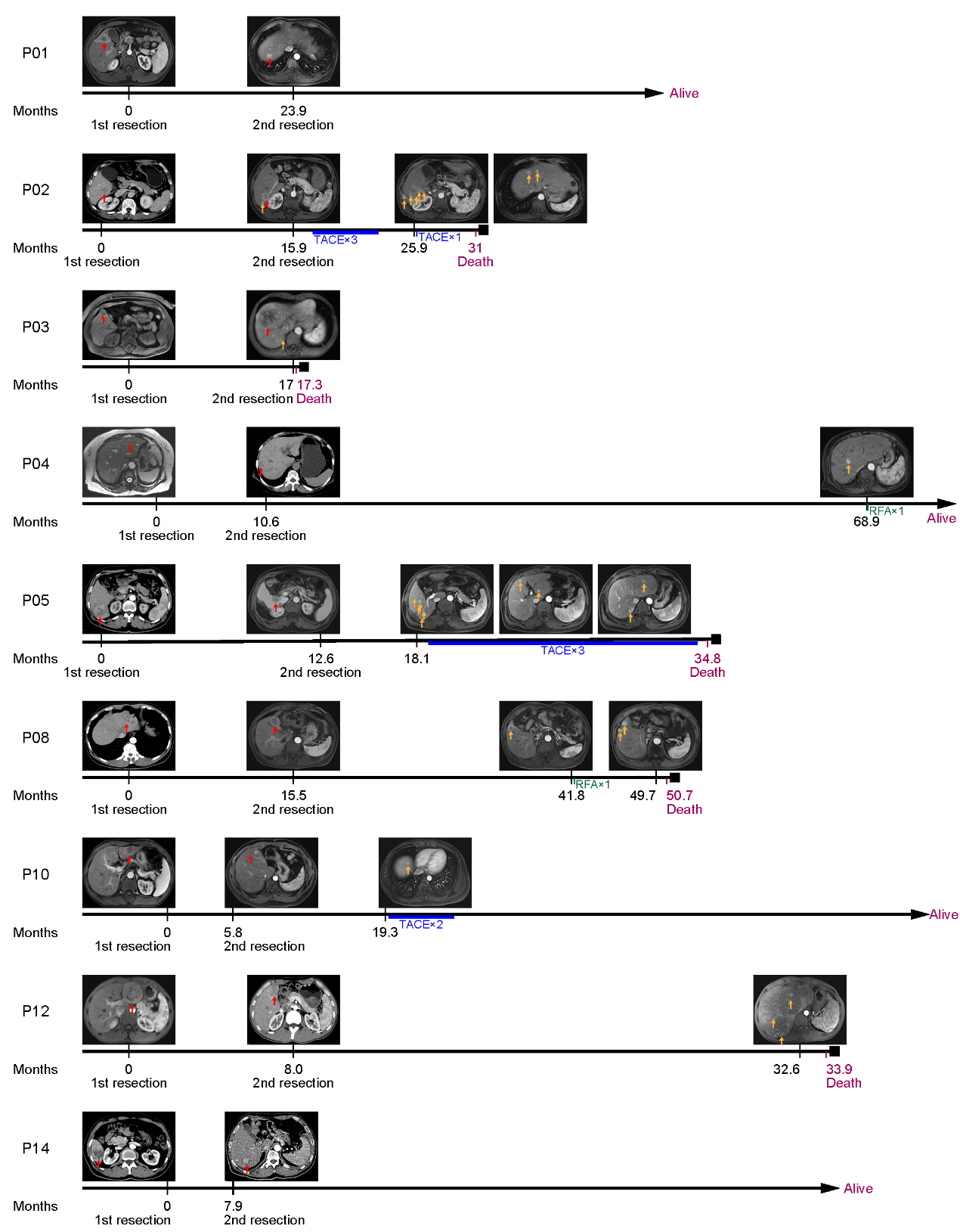


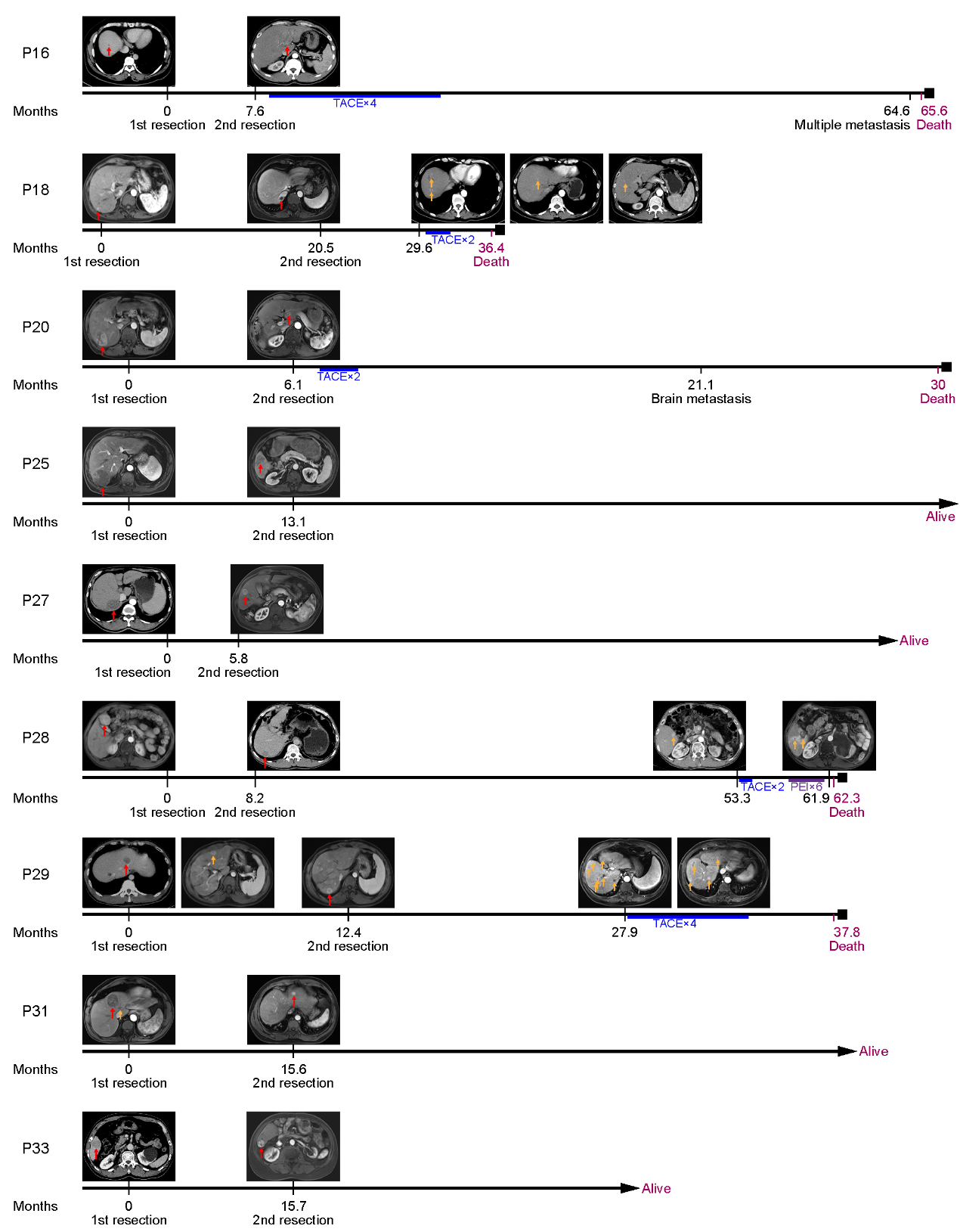


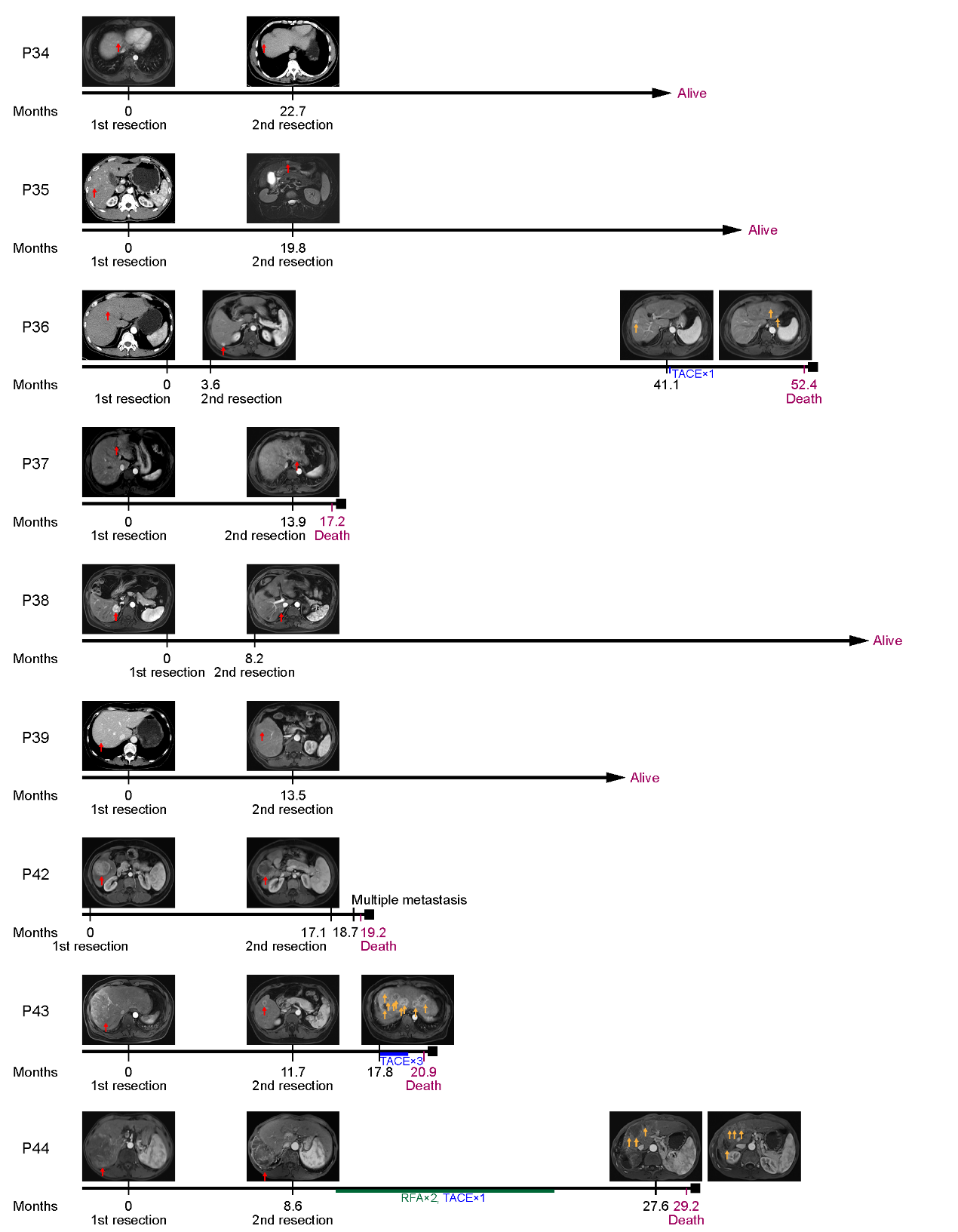


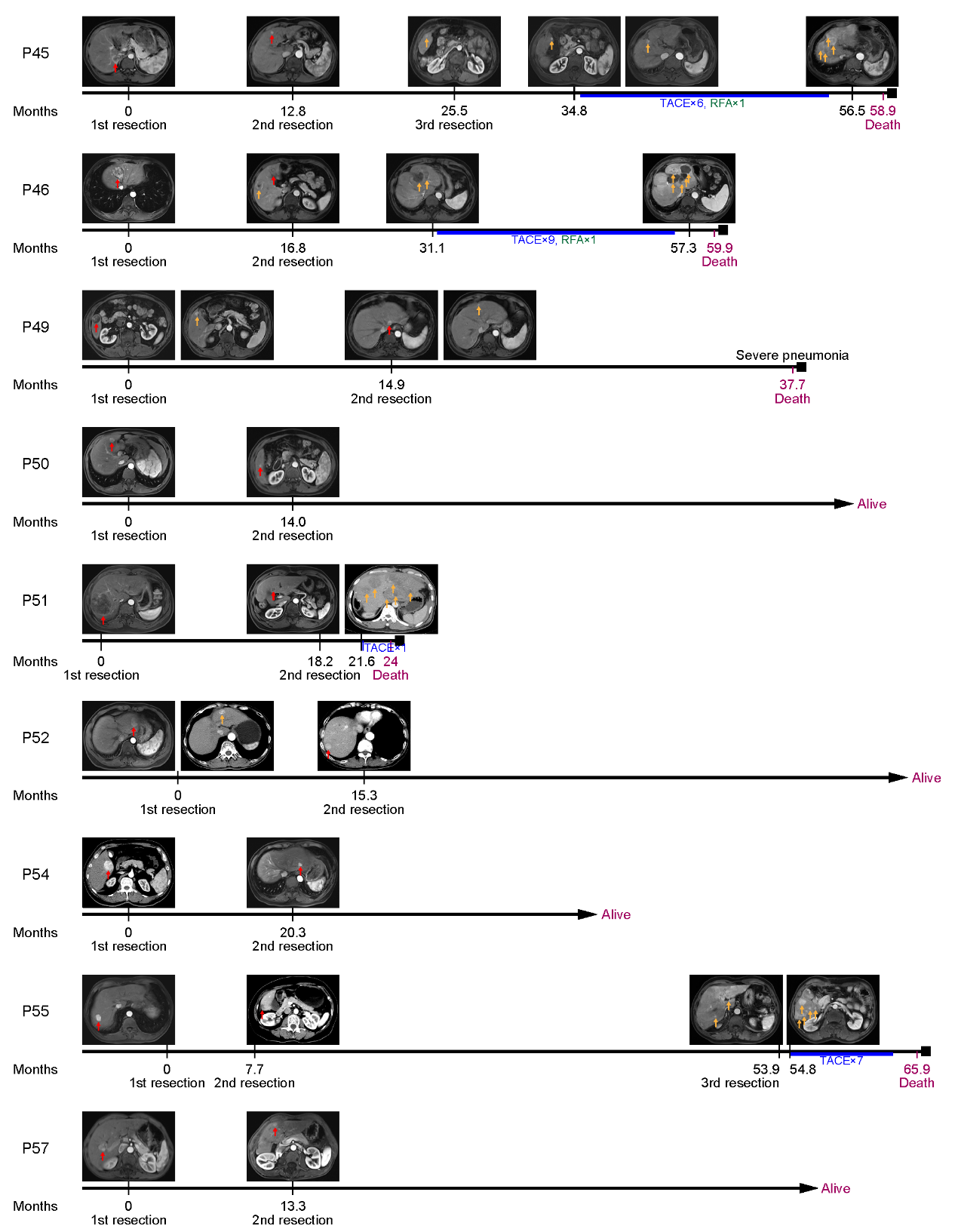


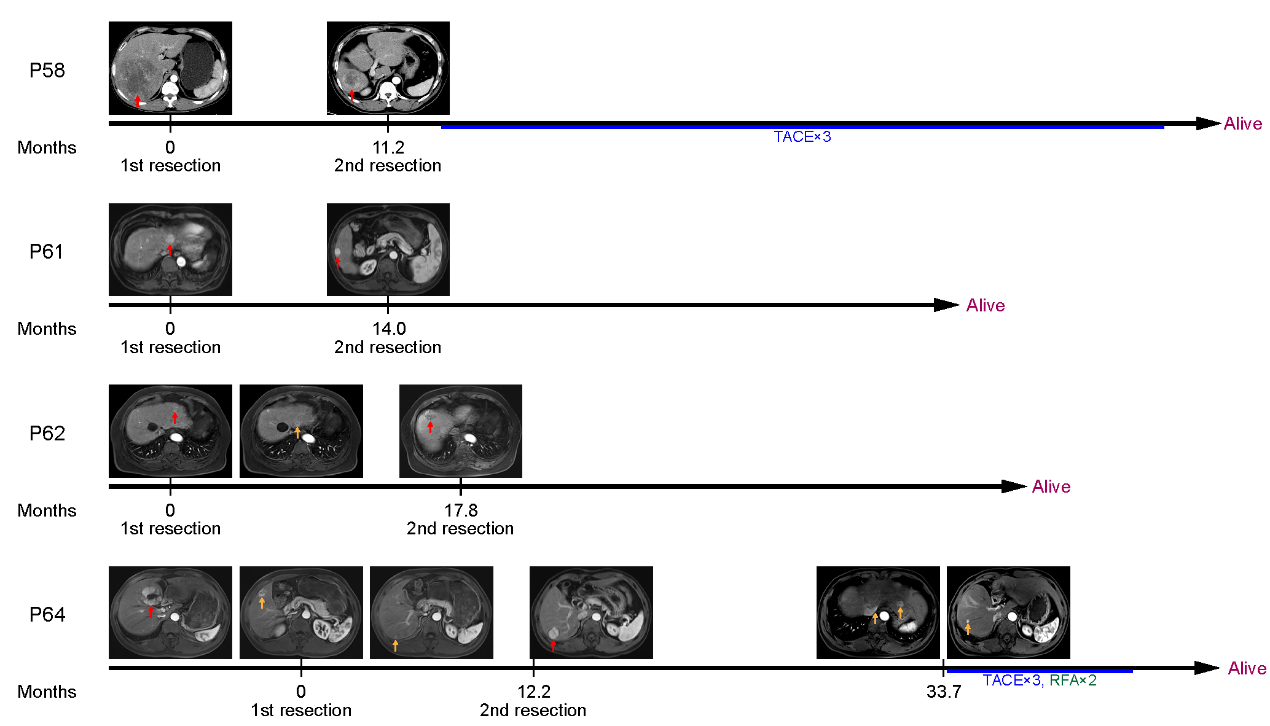


**Supplementary Fig. 3** Timeline of clinical history of 40 primary HCCs with early recurrence. Representative CT/MRI images of each tumor are shown above. The red arrows indicate the locations of tumors involved in WGS; the orange arrows indicate the locations of tumors not involved in WGS.


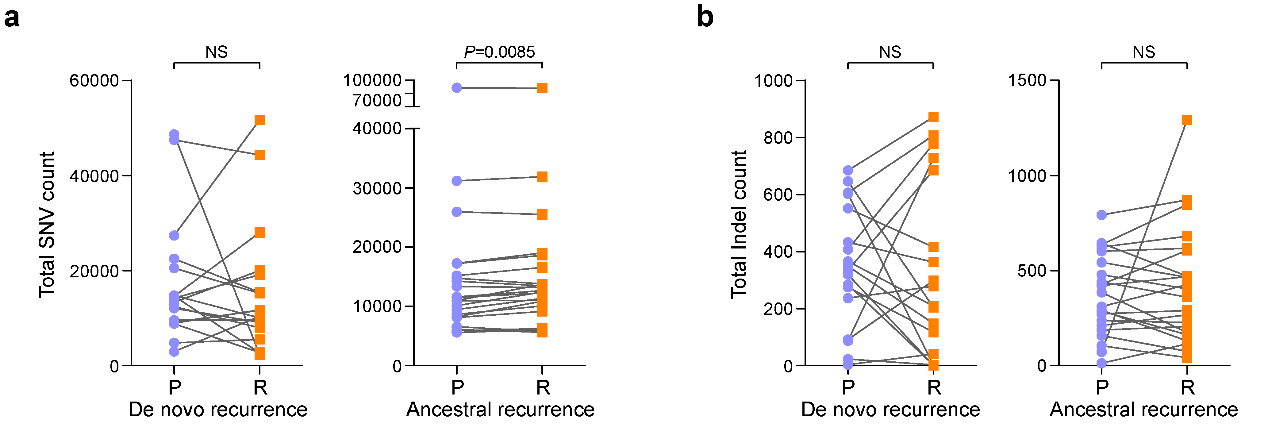


**Supplementary Fig. 4 a** Comparison of total somatic SNV count and (**b)** Indel count between primary tumors and recurrent tumors in patients with de novo recurrence or ancestral recurrence.


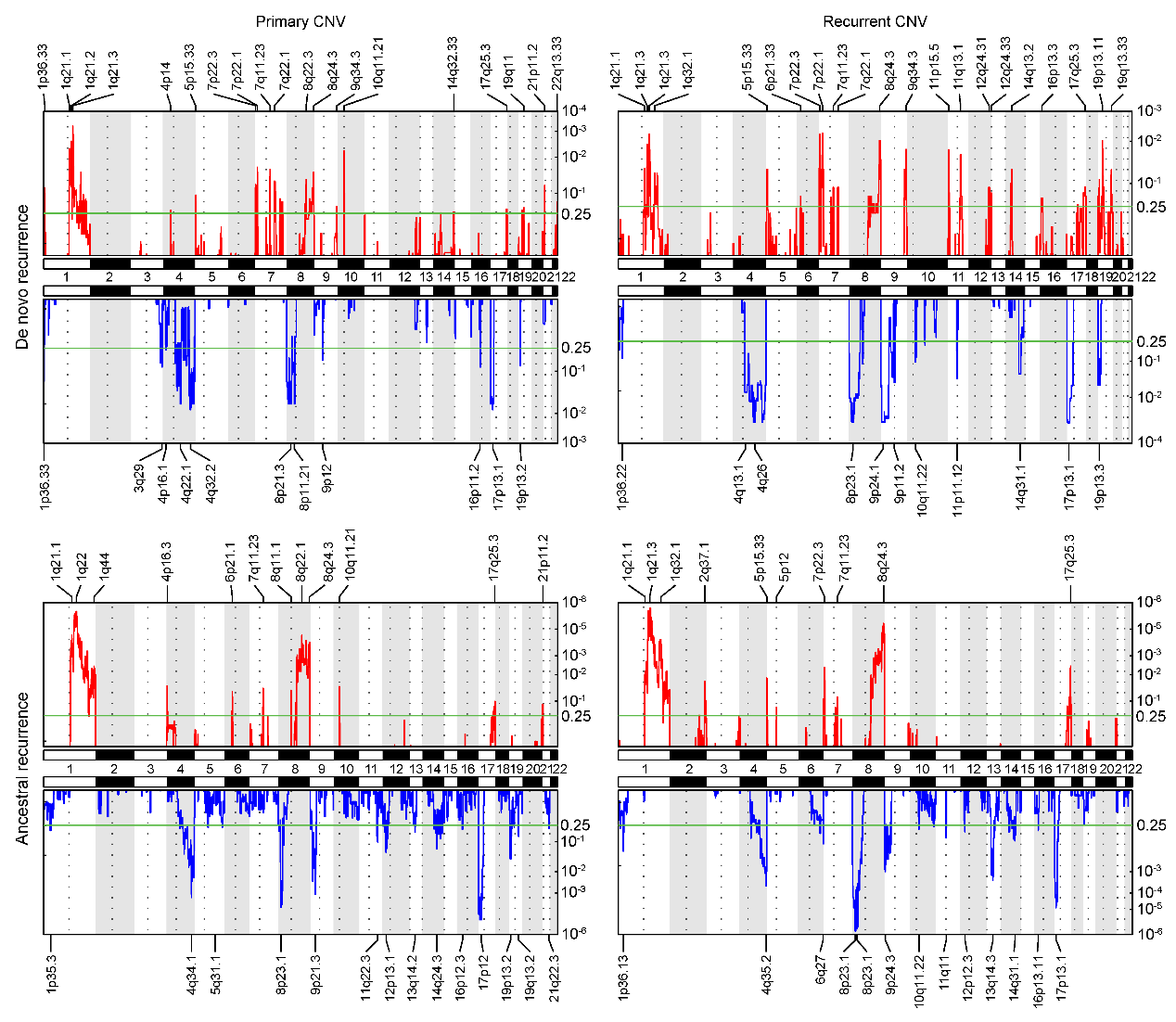


**Supplementary Fig. 5** GISTIC analysis revealed the whole-genome distribution of copy-number alterations in paired primary (left panel) and early-recurrent (right panel) tumors in patients with de novo recurrence (upper panel) or ancestral recurrence (lower panel). GISTIC q-values (y-axis) for amplifications (red) and deletions (blue) are plotted across the genome (x-axis).


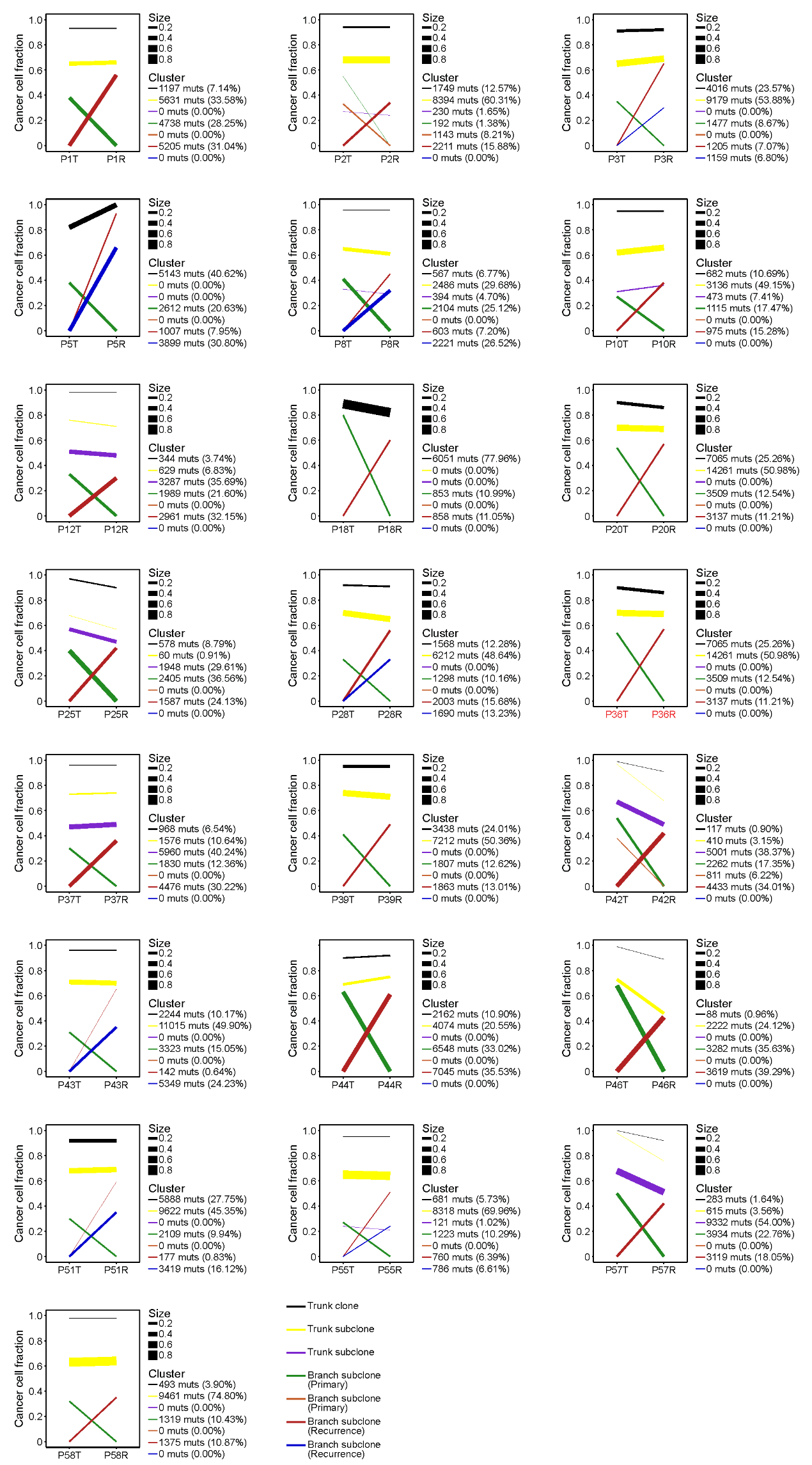


**Supplementary Fig. 6** The mutation clusters detected in the primary tumors and recurrent tumors from 22 patients with ancestral recurrence of HCC. The relationships between clusters in the primary and recurrent tumors are indicated by the lines linking the clusters. Line thickness indicates the relative mutation number in a given patient.


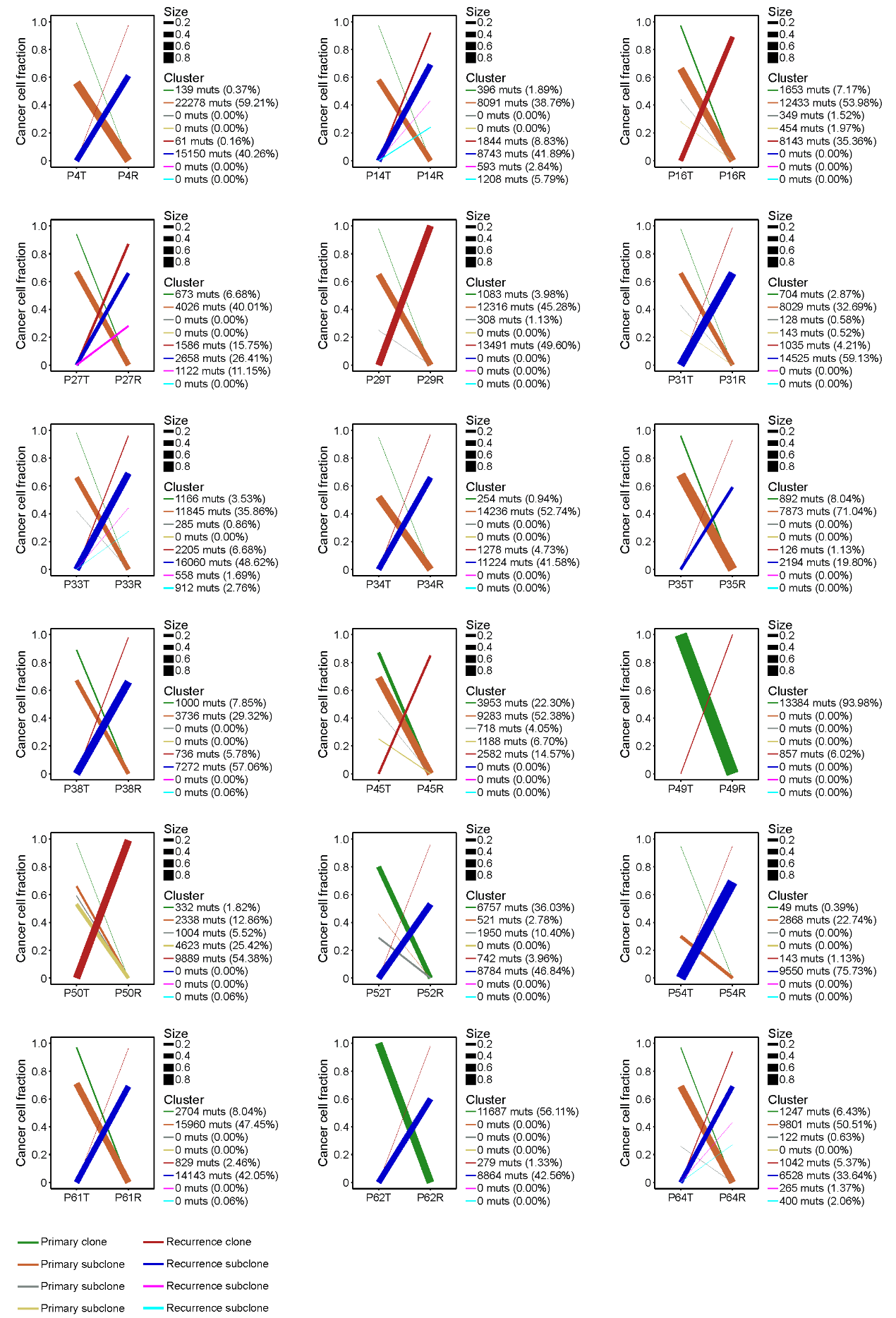


**Supplementary Fig. 7** The mutation clusters detected in the primary tumors and recurrent tumors from 18 patients with de novo recurrence of HCC. The relationships between clusters in the primary and recurrent tumors are indicated by the lines linking the clusters. Line thickness indicates the relative mutation number in a given patient.


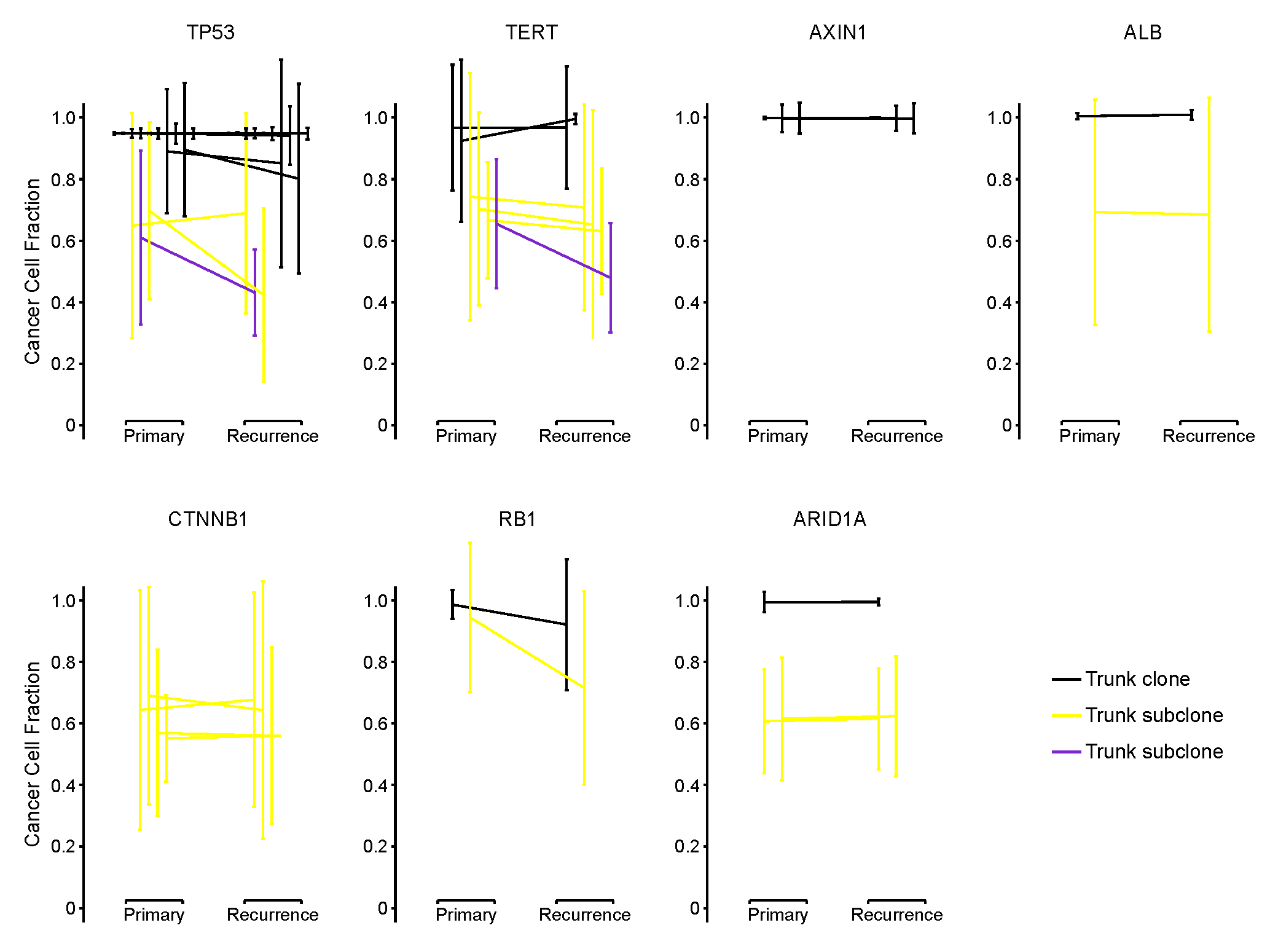


**Supplementary Fig. 8** Comparison (mean CCF with 95% CI) between primary tumors and recurrent tumors for main HCC drivers in 22 patients with ancestral recurrence.


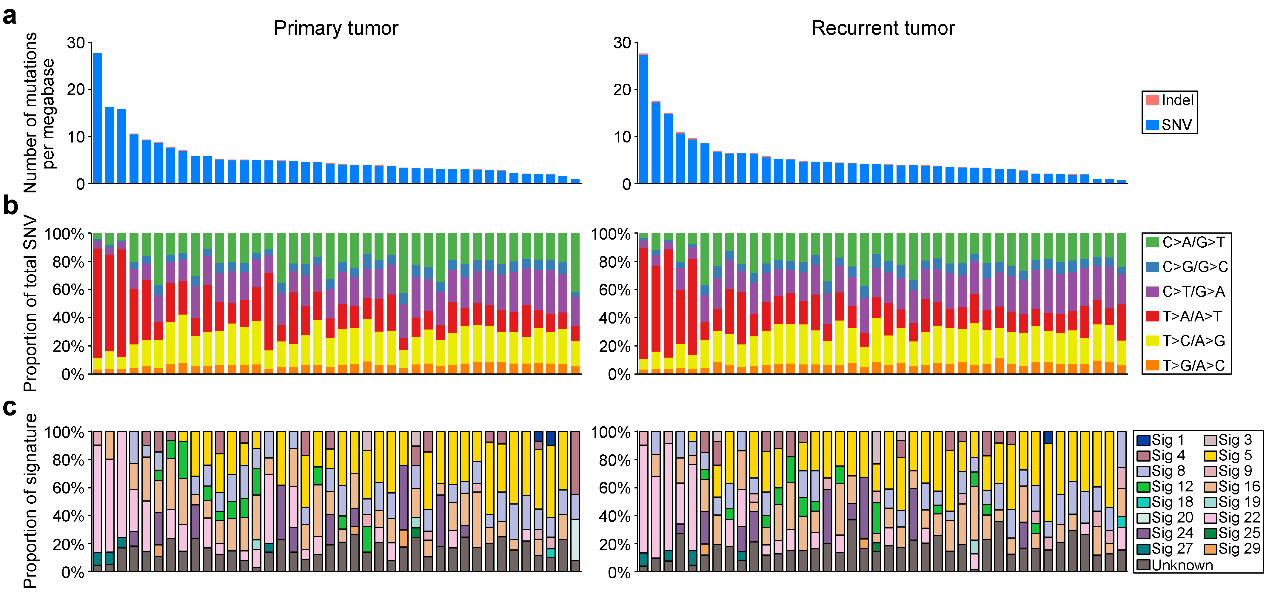


**Supplementary Fig. 9 Mutational spectrum and signatures in primary and early-recurrent HCC. a** Mutational burden in the whole genome across 40 pairs of primary and recurrent HCCs. **b** Distribution of six substitution patterns sorted by the total mutation number. **c** Proportions of signatures observed in each HCC sample.


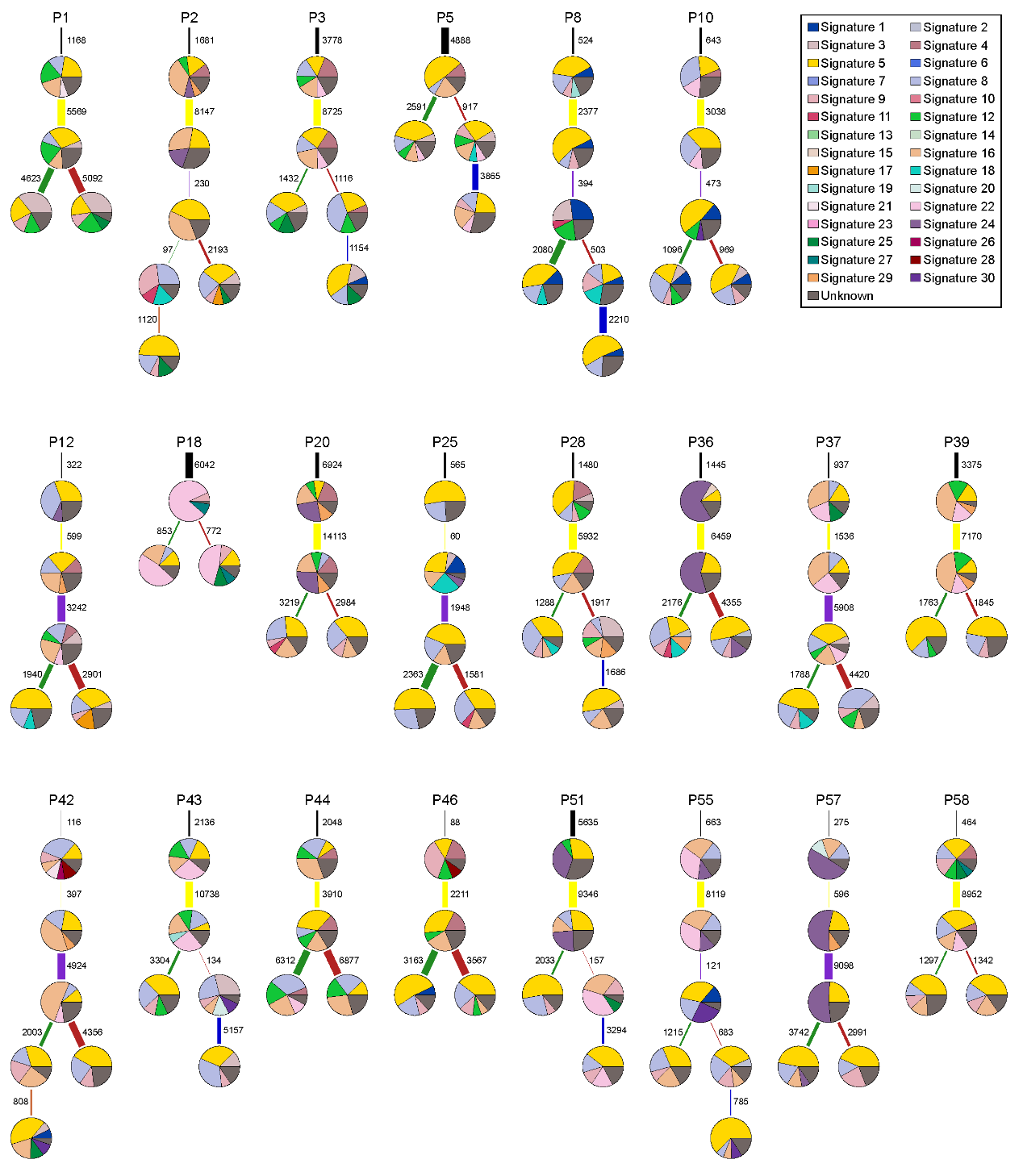


**Supplementary Fig. 10** Pie charts indicating mutational signature evolution during tumor early recurrence based on subclonal architecture in 22 patients with HCC ancestral recurrence, corresponding to Figure 3A. The value on each branch represents the number of SNVs attributed to each pie involved in the extraction of mutational signatures.


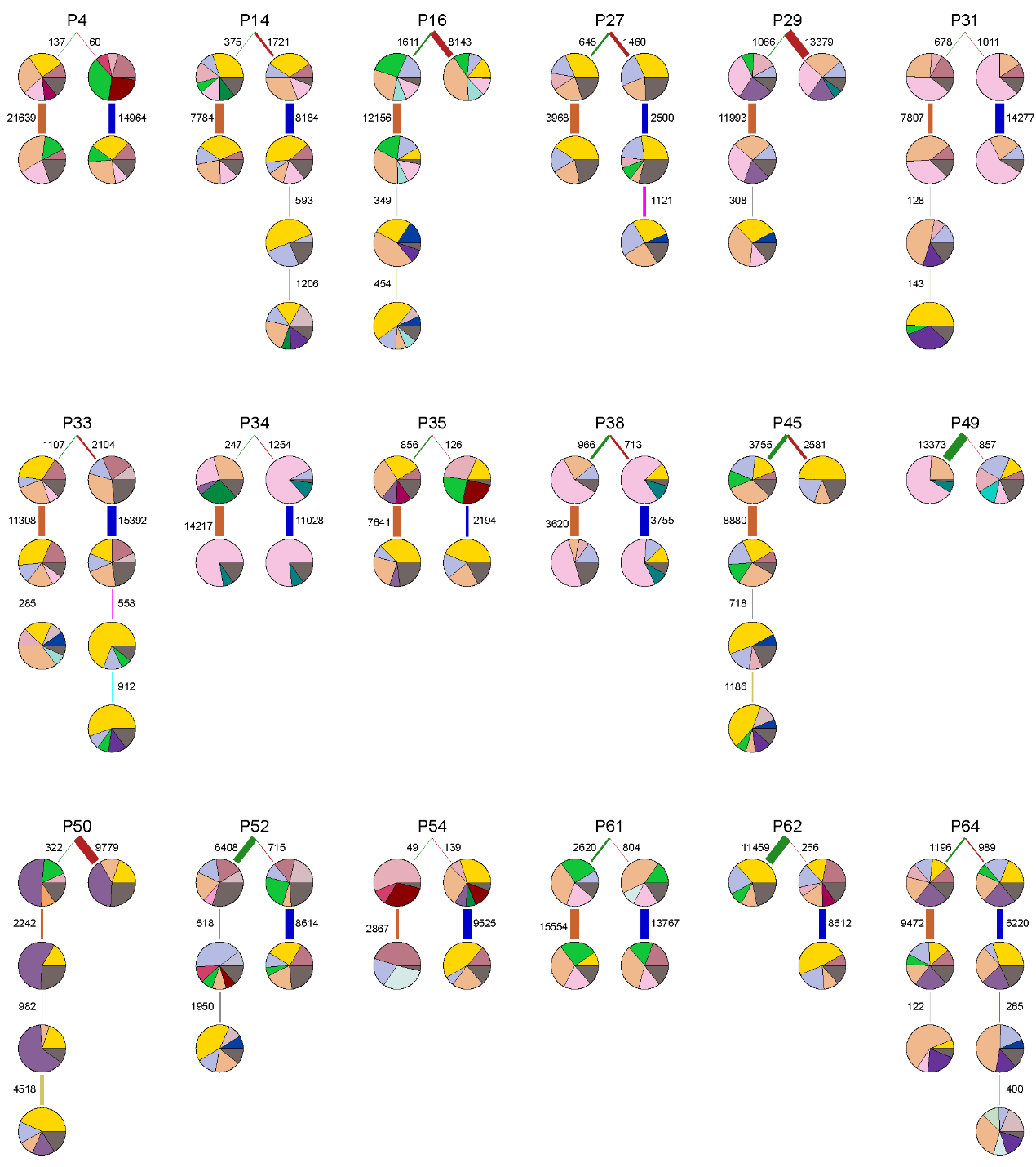


**Supplementary Fig. 11** Pie charts indicating mutational signature evolution during tumor early recurrence based on subclonal architecture in 18 patients with HCC de novo recurrence , corresponding to Figure 4A. The value on each branch represents the number of SNVs attributed to each pie involved in the extraction of mutational signatures.


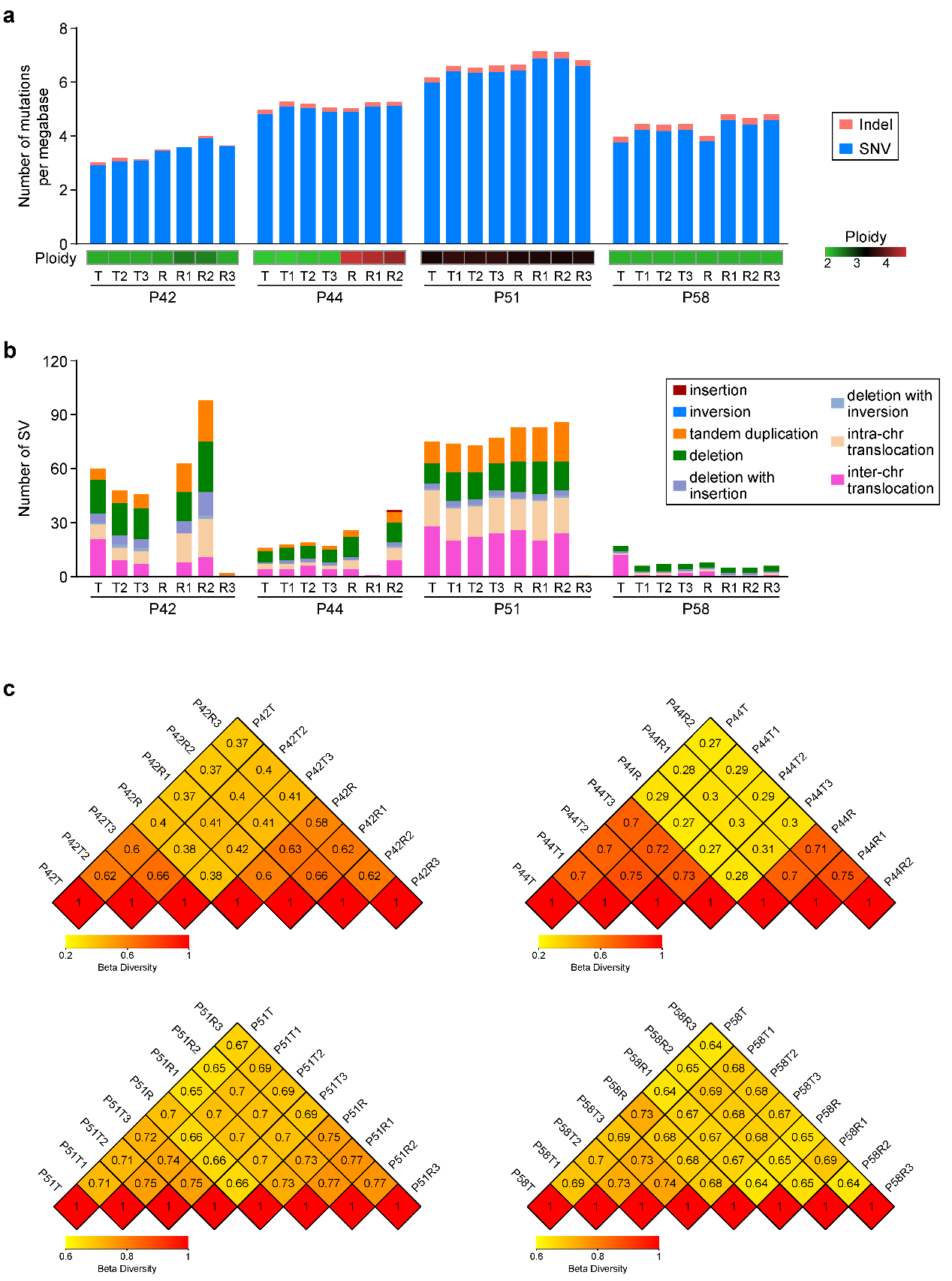


**Supplementary Fig. 12 Genomic landscape of paired primary and early-recurrent tumors (total of 30 tumor samples) from four patients with HCC ancestral recurrence revealed by multi-region whole-genome sequencing. a** Mutational burden of 30 tumor samples from four patients with HCC ancestral recurrence. **b** The number of structural variations in 30 tumor samples from four patients with HCC ancestral recurrence. **c** The Jaccard similarity coefficient between every paired region from primary and recurrent tumors in four patients with HCC ancestral recurrence. Larger value means more similarity and less heterogeneity between two different regions.


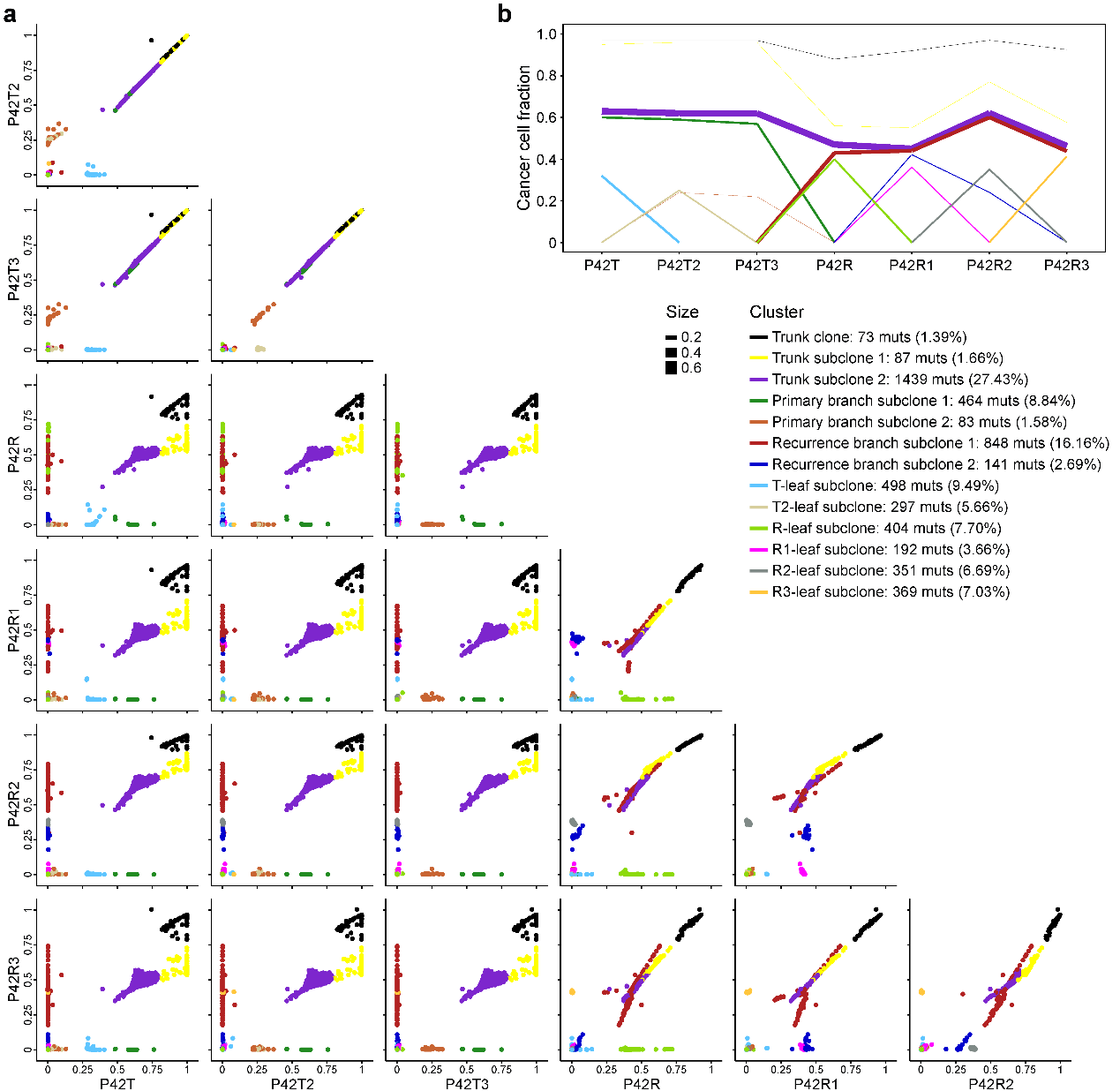


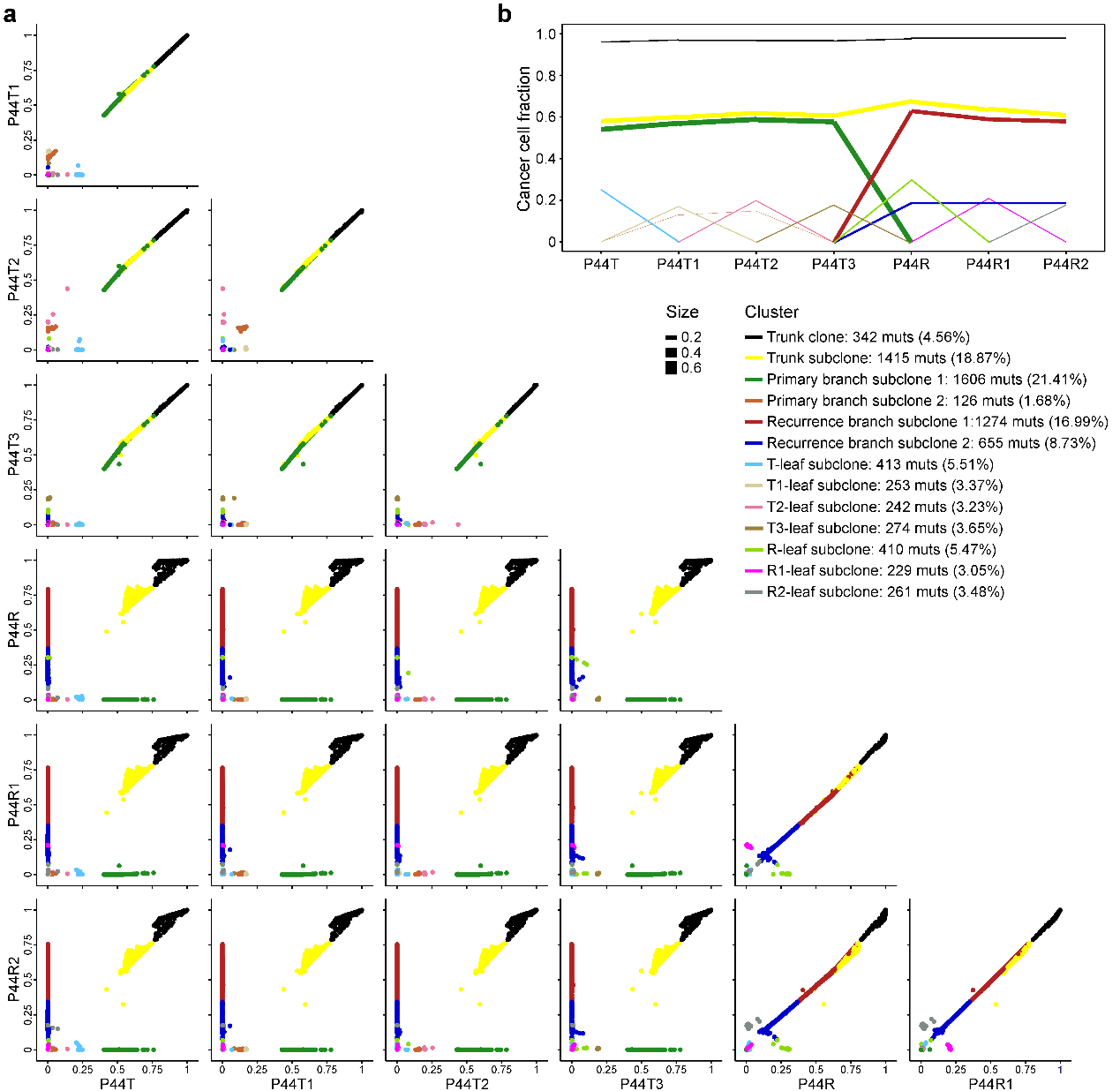


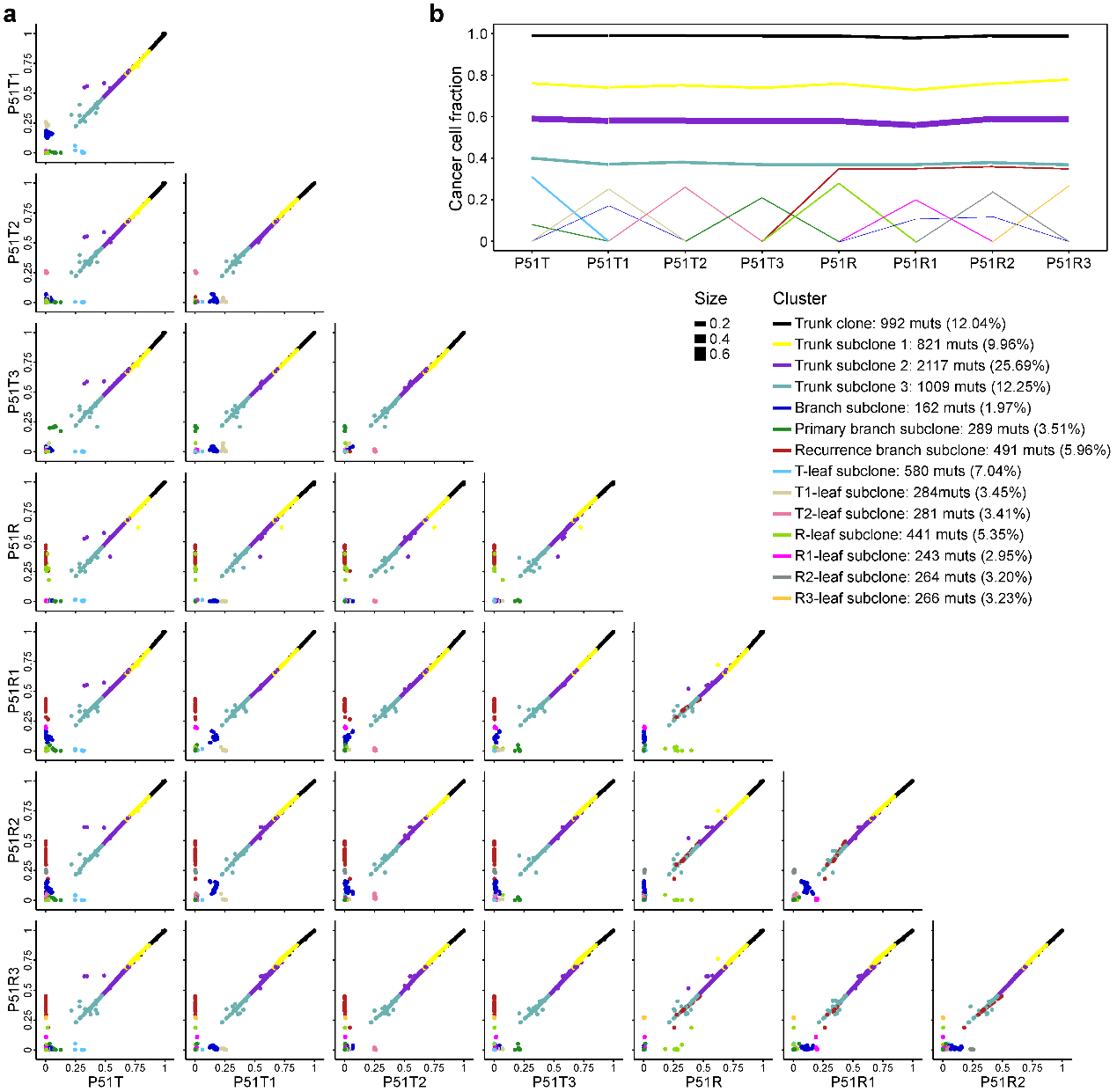


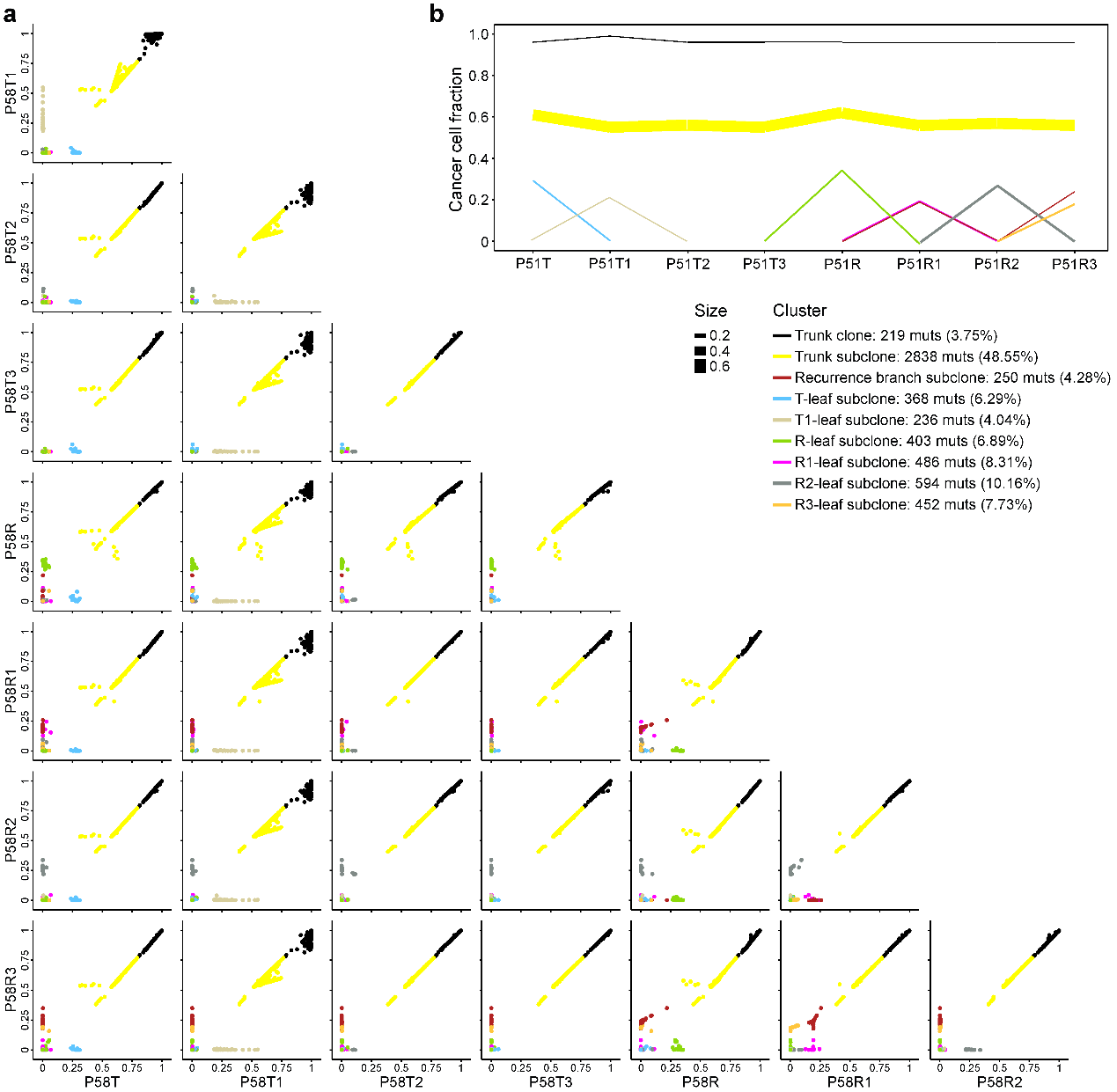


**Supplementary Fig. 13–16 The approaches used to construct the subclonal architectures and phylogenetic trees represented in Figure 6. a** Scatterplot of CCF for somatic mutations in genic region in every paired region of primary and recurrent tumors from four multi-regional sampled patients with HCC ancestral recurrence. **b** The mutation clusters detected in every region of the primary and recurrent tumors from four patients with HCC ancestral recurrence. The relationships between clusters in different regions of the primary and recurrent tumors are indicated by the lines linking the regions. Line thickness indicates the relative mutation number in a given patient.


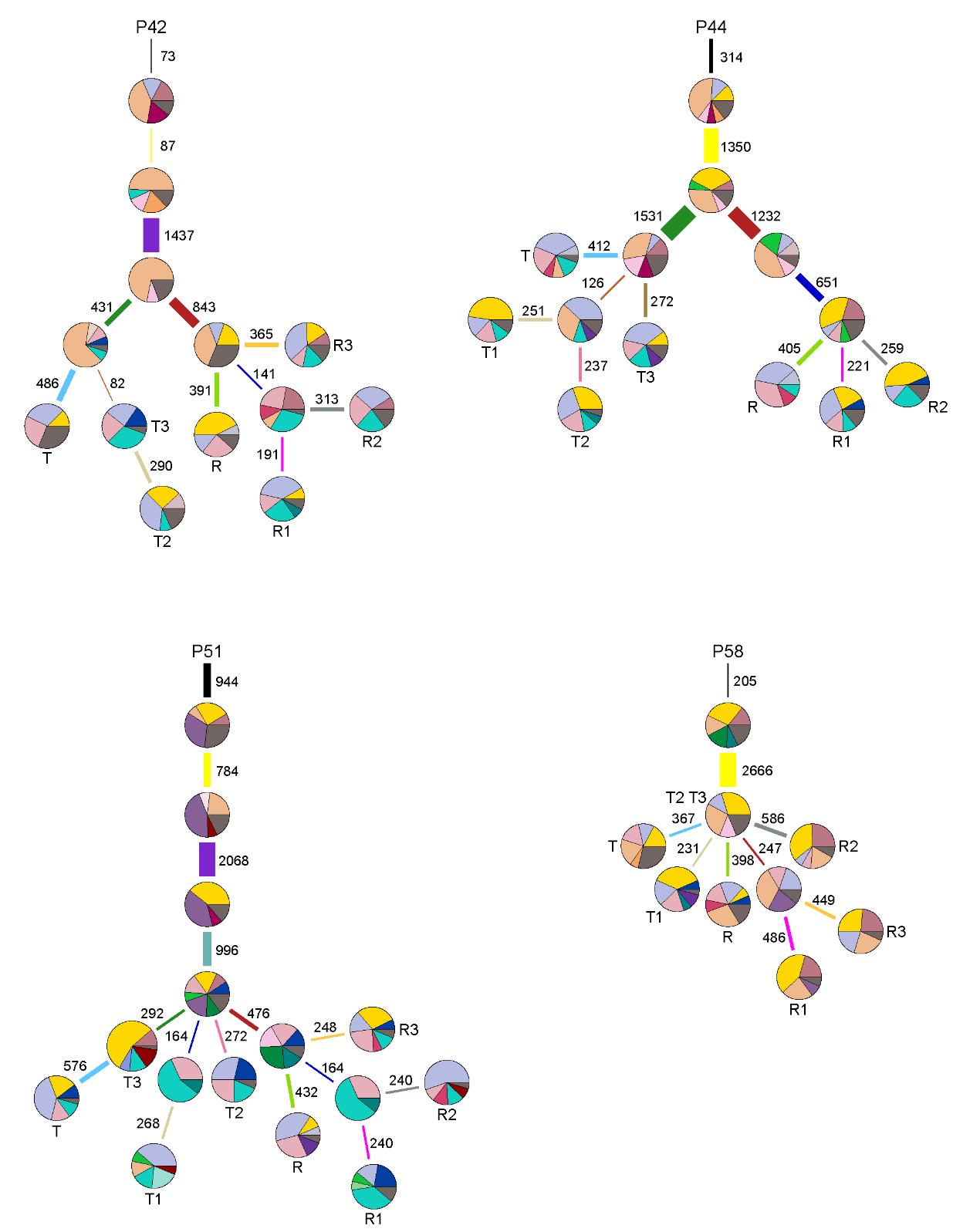


**Supplementary Fig. 17** Pie charts indicating mutational signature evolution during tumor early recurrence based on subclonal architecture in four patients with HCC ancestral recurrence. The value on each branch represents the number of SNVs attributed to each pie involved in the extraction of mutational signatures.


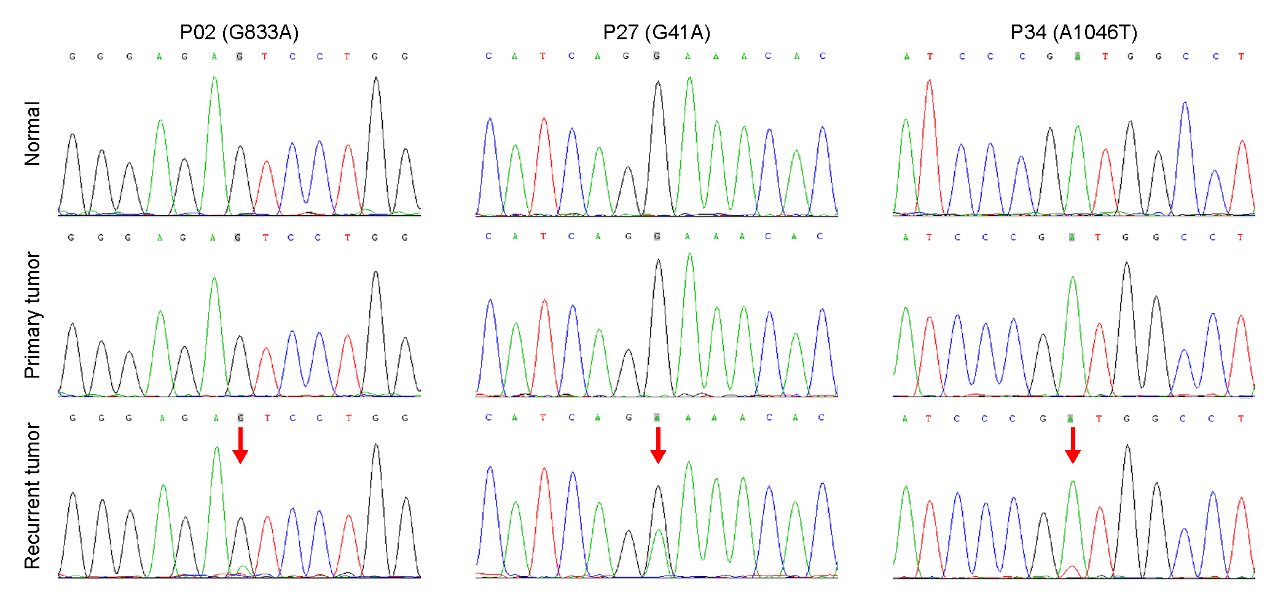


**Supplementary Fig. 18** The Sanger sequencing trace files of *BCL9* mutations in the primary and recurrent tumor samples. The somatic nature of the mutations was determined by comparisons of the sequenced regions between normal and tumor tissues. Specific mutations are listed and highlighted by arrows.


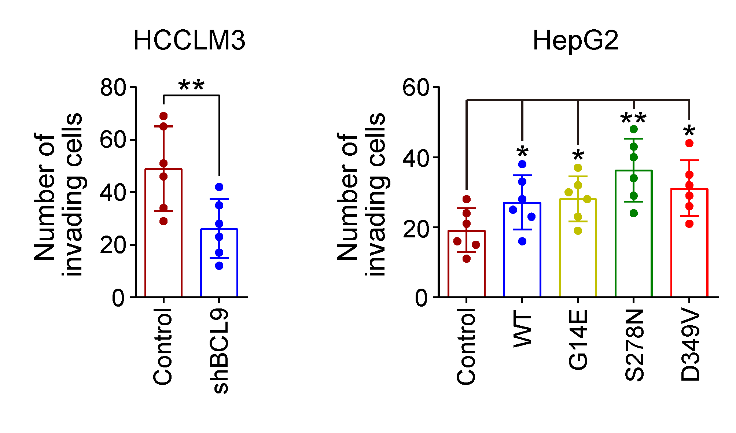


**Supplementary Fig. 19** Standardized quantification of the invasion assay with the cell growth assay.


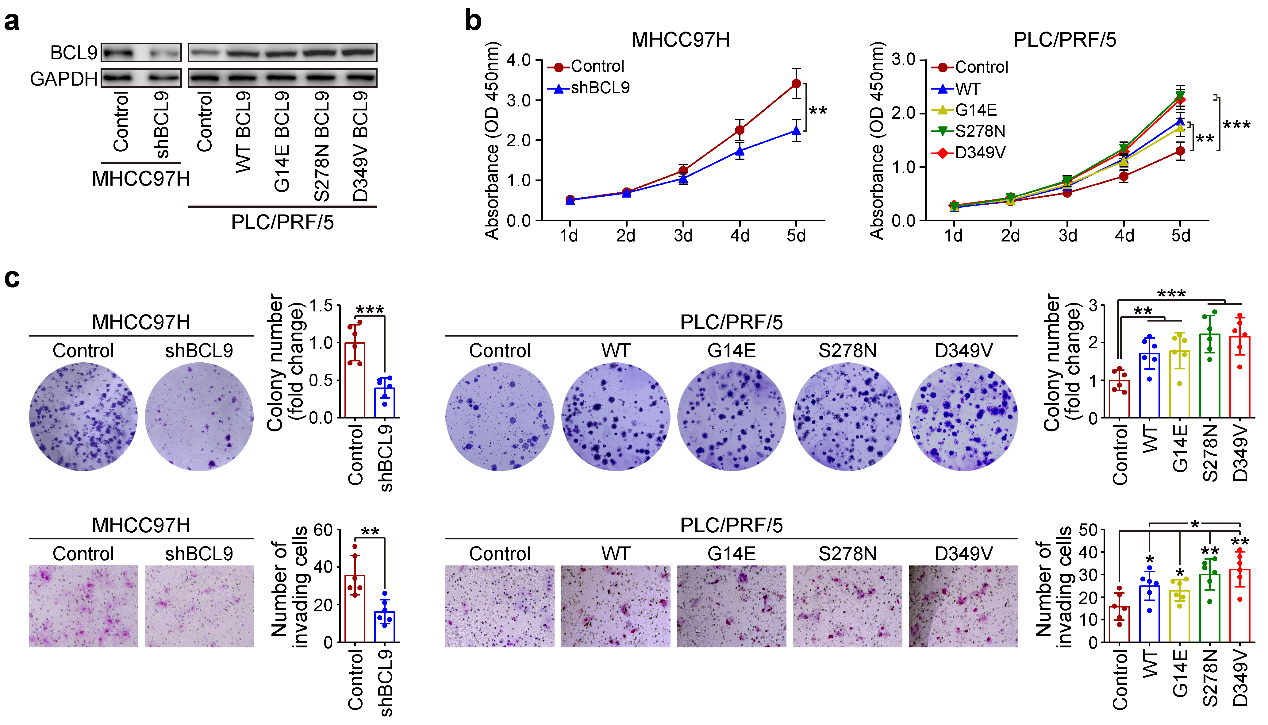


**Supplementary Fig. 20 Oncogenic role of BCL9 in HCC. a** BCL9 expression examined by western blot in stably transfected cells. **b** Proliferation of MHCC97H cells after BCL9 knockdown and of PLC/PRF/5 cells expressing wild-type or mutant BCL9 compared with that of controls, ***P*<0.01, ****P*<0.001. **c** Colony formation and invasion of MHCC97H cells after BCL9 knockdown and of PLC/PRF/5 cells expressing wild-type or mutant BCL9 compared with that of controls. The bar graphs illustrate the quantification of the assay results, **P*<0.05, ***P*<0.01, ****P*<0.001.


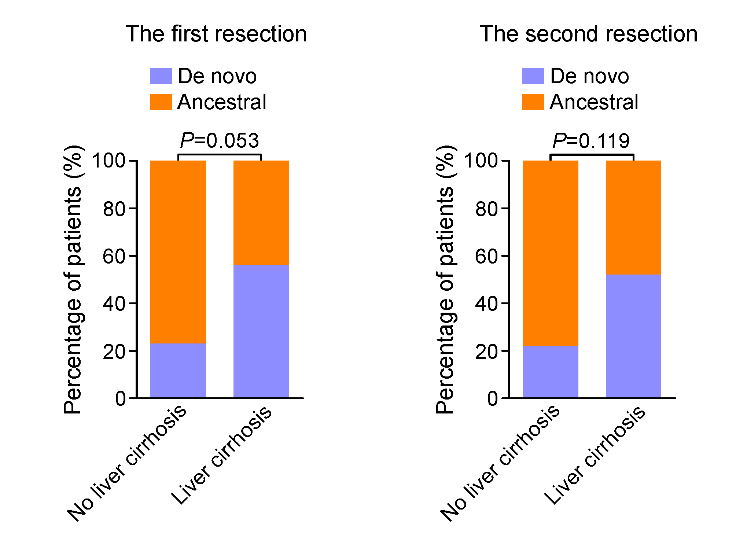


**Supplementary Fig. 21** The association between liver cirrhosis and the recurrence patterns in the WGS cohort totaling 40 HCC patients. Chi-square test was used.

**Reference**

1. Zhou, S.L. et al. CXCR2/CXCL5 axis contributes to epithelial-mesenchymal transition of HCC cells through activating PI3K/Akt/GSK-3beta/Snail signaling. *Cancer Lett* **358**, 124-135 (2015).

2. Cai, J. et al. MicroRNA-374a activates Wnt/beta-catenin signaling to promote breast cancer metastasis. *J Clin Invest* **123**, 566-579 (2013).

3. Tian, J. et al. New human hepatocellular carcinoma (HCC) cell line with highly metastatic potential (MHCC97) and its expressions of the factors associated with metastasis. *Br J Cancer* **81**, 814-821 (1999).

4. Zhou, S. et al. Tacrolimus enhances the invasion potential of hepatocellular carcinoma cells and promotes lymphatic metastasis in a rat model of hepatocellular carcinoma: involvement of vascular endothelial growth factor-C. *Transplant Proc* **43**, 2747-2754 (2011).

5. Zhou, Z.J. et al. HNRNPAB induces epithelial-mesenchymal transition and promotes metastasis of hepatocellular carcinoma by transcriptionally activating SNAIL. *Cancer Res* **74**, 2750-2762 (2014).
